# Systems-level understanding of ethanol-induced stresses and adaptation in *E. coli*

**DOI:** 10.1038/srep44150

**Published:** 2017-03-16

**Authors:** Huansheng Cao, Du Wei, Yuedong Yang, Yu Shang, Gaoyang Li, Yaoqi Zhou, Qin Ma, Ying Xu

**Affiliations:** 1 Computational Systems Biology Laboratory, Department of Biochemistry and Molecular Biology, and Institute of Bioinformatics, the University of Georgia, Athens, GA 30602, USA; 2 BioEnergy Science Center, Oak Ridge National Laboratory, Oak Ridge, TN 37831, USA; 3 College of Computer Science and Technology, Jilin University, Changchun, 130012, China; 4 Institute for Glycomics and School of Information and Communication Technology, Griffith University, Parklands Dr., Southport, QLD 4222, Australia; 5 Department of Agronomy, Horticulture and Plant Science, South Dakota State University, Brookings, SD 57007, USA; 6 BioSNTR, Brookings, SD, 57007, USA

## Abstract

Understanding ethanol-induced stresses and responses in biofuel-producing bacteria at systems level has significant implications in engineering more efficient biofuel producers. We present a computational study of transcriptomic and genomic data of both ethanol-stressed and ethanol-adapted *E. coli* cells with computationally predicated ethanol-binding proteins and experimentally identified ethanol tolerance genes. Our analysis suggests: (1) ethanol damages cell wall and membrane integrity, causing increased stresses, particularly reactive oxygen species, which damages DNA and reduces the O_2_ level; (2) decreased cross-membrane proton gradient from membrane damage, coupled with hypoxia, leads to reduced ATP production by aerobic respiration, driving cells to rely more on fatty acid oxidation, anaerobic respiration and fermentation for ATP production; (3) the reduced ATP generation results in substantially decreased synthesis of macromolecules; (4) ethanol can directly bind 213 proteins including transcription factors, altering their functions; (5) all these changes together induce multiple stress responses, reduced biosynthesis, cell viability and growth; and (6) ethanol-adapted *E. coli* cells restore the majority of these reduced activities through selection of specific genomic mutations and alteration of stress responses, ultimately restoring normal ATP production, macromolecule biosynthesis, and growth. These new insights into the energy and mass balance will inform design of more ethanol-tolerant strains.

Ethanol generation by bacteria represents a promising approach to biofuel production at an industrial scale. Substantial research efforts have been invested into strain optimization for the cost-effective biofuel production by microbes. One of the key challenges in realizing this lies in the toxicity of the produced ethanol to the host cells, which makes the bacteria sick with reduced ethanol production and viability. Previous studies have identified cell membrane damage^1^,^2^,^3^, reduced cross-membrane proton potential^4^,^5^, and reduced peptidoglycan cross-linking in cell wall^2^,^6^ as the main reasons for the reduced productivity and viability of the affected cells. To understand the detailed mechanisms, a number of ethanol-response regulons such as ArcA and FNR along with numerous ethanol-tolerance genes have been identified using omics techniques and extensively studied^7^,^8^,^9^,^10^,^11^,^12^. However, the overall understanding about the impact of ethanol-induced stress, short-term response and long-term adaptation remains to be somewhat fragmented, which is yet to offer very informative guidance to engineering highly ethanol-tolerant biofuel producers. One important reason is that there is a lack of a systems-level understanding about the impact of ethanol stress, specifically how different impacts are related to each other and which may be the root causes of the observed ethanol-induced cellular level alterations. For example, we are yet to fully understand which cellular subsystems are directly affected by ethanol, which are the results of stress responses, and how the reduced functionalities of these subsystems are compensated by other subsystems. In addition, information is still lacking regarding which macromolecules can directly bind with ethanol, hence having their functions disrupted at the cellular level.

We present a computational study of published microarray gene-expression data in the public domain of *E. coli* cells with ethanol treatment *versus* ethanol-free controls in the non-ethanol-adapted (NEA) samples, aiming to derive how ethanol affects the normal functions of the cells. We have also examined gene-expression and genomic data of *E. coli* cells that are ethanol-adapted (EA) after 2,496 hours of evolution, to elucidate how the cells have adapted to the ethanol-induced stress and regained their viability. Our overall findings include: (1) ethanol disrupts and damages cell wall and membranes, resulting in increased oxidative and hypoxic stresses among others; (2) decreased cross-membrane proton potential due to membrane damage, along with hypoxia, leads to reduced ATP production by aerobic respiration, which drives the cells to turn up other means for ATP production, namely anaerobic respiration and fermentation; (3) the reduced ATP production results in reduction in macromolecular biosynthesis and cell proliferation, and triggers increased catabolism of carbohydrates and fatty acid oxidation for energy production; (4) ethanol can directly bind with hundreds of proteins, including transcription factors, which alters their normal functions; (5) these impacts are functionally linked with each other, resulting in reduced viability and growth in the affected cells; and (6) ethanol-adapted *E. coli* cells have restored some key activities through selection of specific mutations and changes in stress response, leading to restored ATP production and macromolecule biosynthesis, reduced stresses, repaired membranes, and ultimately regained cellular viability and growth. Based on these findings, we have constructed a model for ethanol stress and response as well as adaptation by *E. coli* cells.

The main contribution of this study includes: (i) a systems-level model for ethanol-induced stresses in *E. coli* cells as well as for adaptation to the stresses, which integrate new findings and previous observations; (ii) cell membrane damage and the stress-induced ROS (reactive oxygen species) production may be at the root of the observed changes as outlined above; and (iii) a number of new observations regarding how ethanol may contribute to the reduced cellular viability and growth, as well as how the adapted cells overcome the ethanol-induced adversity.

## Results

Computational analyses of transcriptomic and genomic data in the public domain (Supplementary Table S1) combined with ethanol tolerance genes (Supplementary Table S2) and stress resistance pathways (Supplementary Table S3) were performed to infer how ethanol-induced stresses impact *E. coli* cells at a systems level and how the affected cells adapt to the stresses.

### Impacts of ethanol on NEA samples and cellular responses

**Figure 1:**
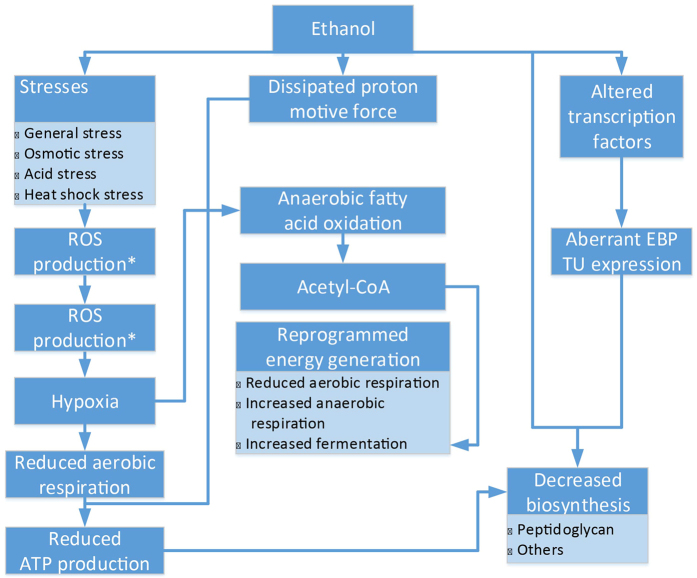
Impact of ethanol on the NEA cells. ROS: reactive oxygen species; EBP: ethanol-binding proteins; and TU: transcription unit. Boxes with asterisks are inferred based on information collected through literature search.

We present here the identified impacts of ethanol and corresponding responses in *E. coli* cells, which are summarized in Fig. 1.

#### Multiple stresses are observed along with increased ROS level

We first examined the types of stress responses to ethanol treatment in the NEA samples. Known responses to acid and osmotic stress, envelope stress, and heat-shock stress were all observed in our results, with varying degrees of up-regulation of the relevant marker genes. For examples, *asr, gadE, gadA*, and *gadB* are up-regulated for acid stress; *osmC, osmB,* and *ompR* for osmotic stress; *ostA, ostB, treB*, and *treC* for trehalose production in resisting the osmotic stress; *rpoE, degP, asnB*, and *opgG* for envelope stress; and *groS, groL, grpE*, and *metA* for heat-shock stress, as shown in Supplementary Fig. S1.

**Figure 2:**
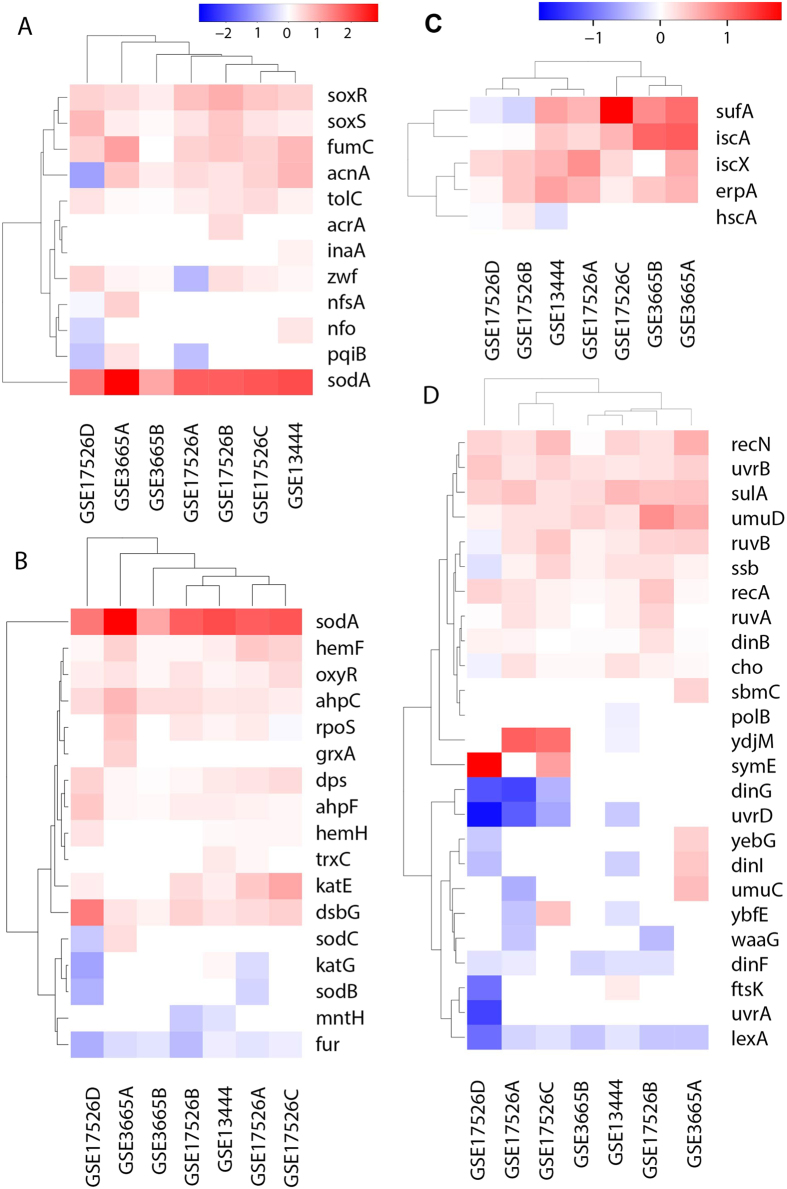
Differential gene expressions in seven pairs of NEA samples. (**A**) SoxRS regulon; (**B**) OxyR regulon; (**C**) iron-sulfur cluster assembly genes; and (**D**) SOS response. In each panel, the x-axis represents the samples used, and the y-axis denotes the proportion of differentially expressed genes out of all. The color definitions for Panels A and B, and for Panels C and D are the same.

Furthermore, we have observed increased response to ROS, which has not received much attention, although it has been found to be induced by ethanol in yeast^13^. Specifically, two key regulons, OxyR and SoxRS, responding to hydrogen peroxide H_2_O_2_ and superoxide (O_2_^−^), respectively, are up-regulated (Fig. 2A and B), including key genes: *sodA, fumC*, and *katE* in at least five out of the seven samples under consideration. Additionally, the expressions of these two regulons correlate with the ethanol concentration, with correlation coefficients 0.53 and 0.49, respectively.

Fenton reaction (Fe^2+^ + H_2_O_2_ -> Fe^3+^ + •HO + OH^−^) has also been observed in *E. coli* when the cells produce substantial amounts of H_2_O_2_, which can react with Fe^2+^ in the iron-sulfur (Fe-S) clusters in proteins^14^,^15^,^16^, producing Fe^3+^, hydroxyl radical (•HO) and hydroxide (OH^−^). Sulfurs in the same cluster can reduce Fe^3+^ back to Fe^2+^ 17, giving rise to another round of Fenton reaction and more damaged iron-sulfur clusters. We found that most genes in Fe-S cluster assembly process are up-regulated (Supplementary Fig. S2C), suggesting that the Fe-S clusters are damaged and hence need to replaced. We also detect a correlation between the Fe-S assembly genes and ethanol concentration, with a correlation coefficient of 0.5. All these provide evidence of Fenton reaction. It is noteworthy that •HO is a most reactive molecule, which can cause substantial damages to nearby molecular structures.

As ROS oxidizes and hence damages macromolecules such as DNA, we also observed an up-regulated SOS response, an error-prone DNA repair system (Fig. 2D). About half of the genes of the SOS response are up-regulated in all the samples, including key genes *recA, recN, sulA*, and *ssb* in at least six of the samples (Fig. 2D).

Since ROS production consumes O_2_ and thus reduces the O_2_ level, hypoxia might occur. The AcrAB two-component system is a switch that dually regulates genes in micro-aerobic conditions^18^. As expected, about 1/3 genes in the ArcAB regulon are up-regulated for anaerobic metabolism, such as *ackA* and *pflB* for fermentation (Supplementary Fig. S3). In addition, a correlation is observed between the SoxRS or OxyR regulon and the ArcAB regulon, with correlation coefficients 0.33 and 0.25, respectively, hence suggesting that the observed hypoxia is related to the increased ROS production.

#### Impact of reduced cross-membrane proton gradient and response

**Figure 3:**
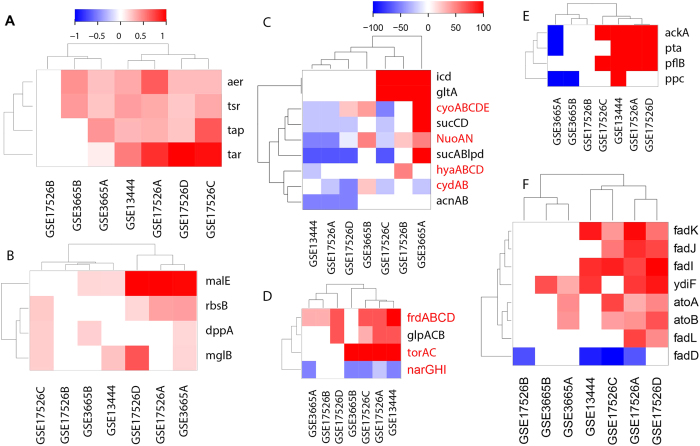
Pathways in response to reduced ATP production in the seven NEA samples. (**A**) energy and chemotaxis sensor genes; (**B**): genes encoding transporters for chemotaxis signal molecules; (**C**–**E**): the proportion of differentially expressed genes encoding enzymes out of all encoding the aerobic respiration pathway (**C**), anaerobic respiration (**D**), and fermentation (**E**) and (**F**) differential expression of genes in fatty acid oxidation pathway. For each panel, the *x*-axis represents samples and the *y*-axis is for genes. Panels A, B and F have the same color definition while panels (**C,D** and **E**) have the same definition, which represents the proportion of genes in each pathway with red and blue representing up- and down-regulation, respectively.

It has been established that ethanol can fluidize cell membrane, leading to decreased cross-membrane proton gradient and thus reduction in ATP production by respiration^4^. Our analysis confirms this in the NEA samples with multiple supporting evidence. First, the major energy sensor genes *aer* and *tsr* are up-regulated in six of seven samples (Fig. 3A). In addition, two other chemotaxis sensor genes *tap* and *tar* are also up-regulated, responding to the signaling molecules imported by four transporters (Fig. 3B). Together, these sensors work synergistically in activating flagellum assembly and cell motility (Supplementary Fig. S4), and genes involved in the two processes are found to be up-regulated for 20 out of the 26 genes (77%) in all the NEA samples (Supplementary Fig. S5). A correlation was also observed between these responses and ethanol concentration, with a correlation coefficient 0.42.

Additional evidence for decreased ATP production includes: (1) DNA replication shows negative correlation with increased ethanol concentration (correlation coefficient: −0.45); (2) major biosynthetic processes, such as cofactor/prosthetic group biosynthesis, phospholipid and amino acid syntheses, all decrease with the increase in ethanol concentration (Supplementary Table S4); and (3) these decreased biosynthetic processes are found to be positively correlated with the decrease in DNA replication (Supplementary Table S5).

**Figure 4:**
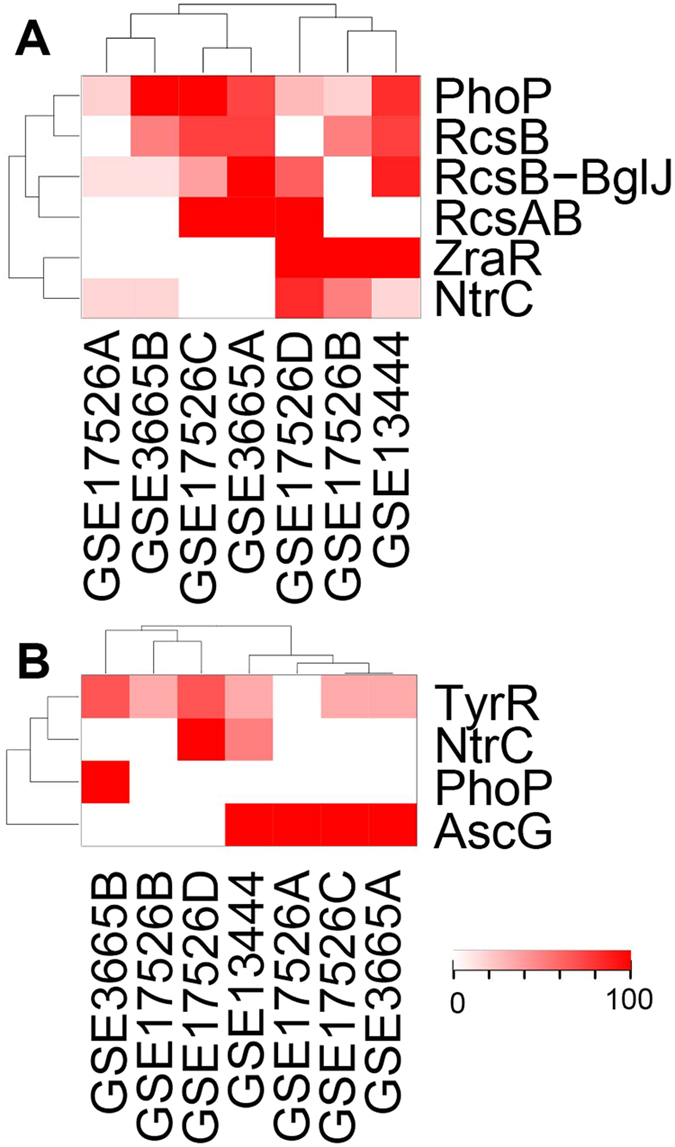
Proportions of differentially regulated TUs by EBP TFs in the NEA samples. The x-axis represents the seven NEA samples and the y-axis is for the EBP TFs. (**A**) activating EBP TFs; and (**B**) repressive EBP TFs.

We then examined cellular responses to the reduced ATP production. We observed that some genes in the electron transport chain are down-regulated with varying degrees of proportions across different samples, such as NADH dehydrogenase (*nuoA-N*), terminal oxidoreductases *cyoABCDE* and *cydAB* (Fig. 3C). Interestingly, two anaerobic oxidoreductases (*frdABCD* and *torA*/*torC*) are up-regulated (Fig. 3D). Some key fermentation enzymes such as pyruvated-formate lyase (*pflB*), phosphate acetyltransferase (*pta*), and acetate kinase (*ackA*) are up-regulated, hence indicating ATP generation through conversion of pyruvate to acetate (Fig. 4C). These changes suggest that with the reduced ATP production by aerobic respiration, the affected cells rely on anaerobic respiratory and fermentation enzymes for ATP production.

Another major response to the reduced ATP production is anaerobic *β*-oxidation of fatty acid in four of the seven samples, which also have up-regulated fermentation activities. The key step of this pathway is to break up long-chain fatty acids to acetyl-CoA that is catalyzed by three anaerobic enzymes (*fadI, fadJ*, and *fadK*), which are all up-regulated in four samples, while one (*fadD*) of the three aerobic counterparts (*fadA, fadB*, and *fadD*) is down-regulated. In addition, the level of *β*-oxidation is found to correlate with the increase in ethanol concentration with a correlation coefficient of 0.49.

#### Ethanol affects protein functions

We have also investigated which proteins may directly bind with ethanol and thus show altered functions by computationally identifying ethanol-binding proteins (EBPs) and examining the possible consequence of ethanol binding. A total of 213 EBPs are predicted, as detailed in Supplementary Table S6, which are involved in 31 biological processes (Supplementary Table S7). Among the most highly enriched processes are oxidation reduction, carbohydrate/polysaccharide biosynthesis, lipid biosynthesis, rRNA/RNA modification, and metabolism of vitamins and cofactors, all of which are consistent with our earlier observation that the affected cells have substantially reduced biosynthesis of macromolecules and increased ROS production. Among all the NEA samples, 78 out of the 213 (37%) EBP genes are up-regulated while 54 (25%) are down-regulated.

We also examined whether ethanol binding may alter protein functions, by comparing the expression profiles of the transcription units (TUs) regulated by EBP transcription factor genes (TFs) with the experimentally validated regulatory relationships as documented in the RegulonDB database^19^. Here, only those TUs with EBP TFs as sole regulators were checked, and 10–100% such TUs were found to be aberrantly expressed (Fig. 4). The total 48 genes in these TUs are involved in multiple biosynthetic processes, such as amine biosynthetic process (Supplementary Table S8). These aberrant TU expressions suggest that functions of these EBPs are likely to be altered by ethanol binding.

#### Reduced peptidoglycan and other macromolecule biosynthesis

Besides interfering with normal cellular processes presented above, ethanol also reduces peptidoglycan biosynthesis among other biosynthetic processes (Supplementary Table S9). One key crosslinking gene *murG* is an EBP (Supplementary Table S6) and is down-regulated in most samples (Supplementary Fig. S6B). Its product, *N*-acetylglucosaminyl transferase, catalyzes the final step of intracellular peptidoglycan crosslinking (Figures S7–S9). Furthermore, all three stages of peptidoglycan biosynthesis (initiation, crosslinking, and maturation) are down-regulated, with 75% (3/4), 92% (12/13), and 76% (16/21) genes down-regulated at each stage, respectively, in six of the seven NEA samples (Supplementary Fig. S6). The down-regulation of the entire pathway correlates negatively with ethanol concentration with a correlation coefficient −0.54. Besides, the expression level of the peptidoglycan biosynthesis has high correlation with other reduced biosynthetic processes as presented in Supplementary Table S9.

We summarize the GO biological processes which are highly enriched by differentially expressed genes in Supplementary Table S10.

### Adaptation in the evolved strains

**Figure 5:**
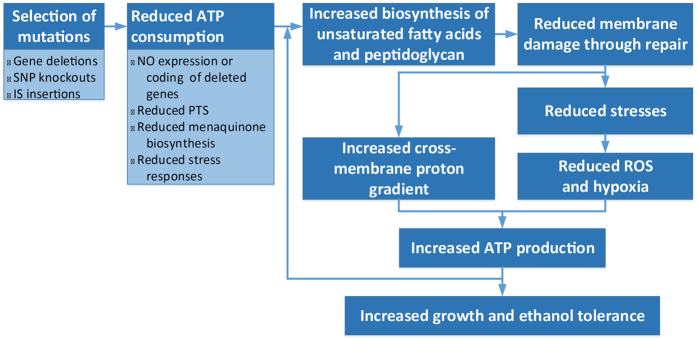
A model for how the evolved NEA strains have adapted to ethanol-induced stresses. Asterisks indicate that our predicted changes are also experimentally reported in literature. Major processes are highlighted in bold. Different colors represent different processes.

Here we address the question: how may the affected cells have adapted to the stresses induced by ethanol ? To answer this question, we collected ethanol tolerance genes that are experimentally identified (Supplementary Table S2) from datasets in the public domain, along with transcriptomic and genomic data of six strains of *E. coli* that have adapted to the ethanol toxicity in parallel^20^,^21^. For each evolved strain, the transcriptomic data were collected at six different time points: h0, h384, h744, h1,224, h1,824 and h2,496. We also examined genomic mutations in these evolved strains. Based on our analyses, we constructed a model for how the EA strains adapt to ethanol at a systems level (Fig. 5).

#### Genomic mutations in the evolved strains

We have examined the mutations accumulated in the ethanol-adapted stains. Of the six strains that evolved from a common parent strain, strain A has a mutant DNA mismatch repair gene *mutS* and hence is deficient in mismatch repair (commonly known as a mutator strain). It has 131 mutations: 125 SNP and 6 indels^20^. A total of 27 mutated genes are found in the other five EA strains: 4 in strain B; 8 in C; and 5 in each of D, E and F. The details of these mutations and their roles in adaptation are provided in Supplementary Table S11. 11 of these genes are involved in gene regulation (Supplementary Fig. S10B), including four global regulator genes: *hns, relA, rpoA* and *rpoC*. Besides, strain C has a large duplication of ~220 Kb^20^, which contains 180 genes (Supplementary Table S12). 96 (53%) of these genes are expressed and up-regulated between h1,224 and h1,824 (Wilcoxon test, *p < *0.01) and continues to h2,496 (Fig. S11A and B); these genes show higher expressions than those in other strains (Supplementary Fig. S11C). Functionally, these up-regulated genes are involved in biosynthetic processes, such as biosynthesis of carbohydrates, polysaccharides and lipopolysaccharides (Supplementary Table S13).

We have also examined 1,623 ethanol tolerance genes that have been experimentally identified either by overexpression or knockout (Supplementary Table S14). 60% (977/1623) of these genes are expressed in all six EA samples, and 49% (480/977) are differentially expressed: 277 up-regulated and 203 down-regulated (Supplementary Fig. S12). Of the 480 genes, 91% showed monotonic changes over time in terms of their differential expressions and match their overexpression or knockout patterns (Supplementary Table S14), i.e., these genes show step-wise up- or down-regulation over the course of evolution as overexpression or knockout (Supplementary Fig. S12). Functionally, most of these genes are involved in transcription, translation, and biosynthesis of vitamins, cofactors, carbohydrates and polysaccharides, and cell wall macromolecules including peptidoglycan (Supplementary Table S15).

#### Reduced non-essential ATP consumption and restored ATP production

We then examined whether the ROS production is still high in the evolved strains by analyzing the level of response to ROS. Compared to the parent strain, response to ROS shows a step-wise decline in mean expression levels of all response genes throughout the evolution (Fig. S13A and D). Most (6/9) genes are down-regulated persistently while only two (*soda* and *ahpF*) are up-regulated. This is consistent with the fact that the majority of the genes in SoxRS and OxyR regulons are consistently down-regulated (Supplementary Fig. S13B, C, E, and F). These down-regulations suggest that ROS level is significantly reduced and the O_2_ level is increased in the EA strains.

To verify that O_2_ level is indeed increased, we examined the expression level of the *nrdD* gene that is essential to anaerobic respiration and is highly repressed by O_2_^22^. Indeed, this gene is dramatically repressed (Supplementary Fig. S14) compared to the parent strain, and its activator *nrdG,* which is also essential for anaerobic respiration^22^, is not expressed. In addition, genes for three key anaerobic enzymes (*narGHI, narZXV*, and *frdABCD*) are not expressed, and the only two expressed genes *narX* and *frdA* are down-regulated progressively during evolution (Supplementary Fig. S15). Furthermore, most fermentation genes are also down-regulated (Supplementary Fig. S16).

**Figure 6:**
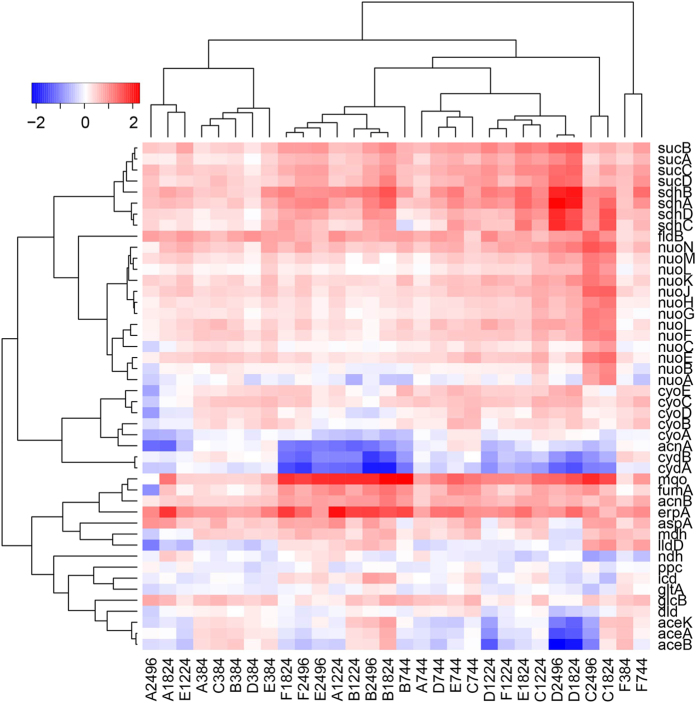
Differentially expressed genes involved in aerobic respiration in EA samples. The *x*-axis is for the six strains (**A–F**) at five time points (384, 744, 1,224, 1,824, and 2,496) and the *y*-axis is for genes involved in aerobic respiration.

Additionally, most genes involved in aerobic respiration are up-regulated (Fig. 6), such as NADH dehydrogenase (*nuoA*-*N*), succinate dehydrogenase (*sdhABCD*), fumarate dehydrogenase (*fumA*), terminal oxidoreductases (*cydAB* and *cyoABCDE*), and lactate dehydrogenase (*ldhA* and *lldD*). Furthermore, we observed (1) no genes encoding energy sensors are expressed in the evolved strains; (2) no genes are expressed for fatty-acid oxidation, except the repressor of this pathway *fadR* and a fatty acid porin *fadL*, which are up-regulated in most EA strains (Supplementary Fig. S17); and (3) 65% (28/43) of biosynthetic processes show positive correlation with DNA replication in the EA samples (Supplementary Table S16), as opposed to the negative correlation between DNA replication and biosynthesis in the NEA samples. All these strongly support that ATP production by aerobic respiration is greatly improved.

We also observed that reduction in non-essential ATP consumption contributes to ATP sufficiency during the adaptive evolution. For example, the *nagE* gene in strain C has a CCG insertion in its coding sequence, causing an insertion of a bulky proline residue right in the middle of a transmembrane helix between residues 282 and 302 in the protein sequence (Supplementary Table S11). The insertion is likely to disrupt the transmembrane channel of the *N*-acetylglucosamine PTS permease, causing a loss of function. This suggests that the entire ATP-consuming PTS system may no longer be needed. This postulation is supported by the fact that only 45% (20/44) of the PTS genes are expressed, of which 55% (11/20) are down-regulated (Supplementary Fig. S18A). Furthermore, seven PTS genes show step-wise decrease in expression level throughout the entire 2,496-hour evolution (Supplementary Fig. S18B). Additionally, the PTS genes show negative correlations with the levels of biosynthetic processes (Supplementary Table S17).

Another energy-saving mutation is in *menC* in strain C, which has an in-frame IS186 (1343 bp) insertion (Supplementary Table S11). As *menC* is involved in the biosynthesis of menaquinone, the quinone for anaerobic respiration, which is not needed as most anaerobic genes are not expressed in the EA samples. Indeed, all five genes in this biosynthesis pathway are down-regulated in all six evolved strains (Supplementary Fig. S19A), and two (*menC* and *menF*) show step-wise decrease in expression level across all six strains (Supplementary Fig. S19B). Overall, a total of 11 genes are deleted or mutated, leading to reduced ATP consumption in all six evolved strains (Supplementary Table S11).

#### Strengthened cell envelope through evolution

Ethanol disrupts peptidoglycan crosslinking in cell wall and even disrupts its physical support for cell structure integrity^6^. We hypothesized that both components of the cell envelope, peptidoglycan and unsaturated fatty acids, of the evolved strains are reinforced through their increased biosynthesis. Indeed, most genes involved in peptidoglycan biosynthesis are consistently up-regulated in all six strains: 75% (3/4) of the initiation-stage genes, 62% (8/13) of the crosslinking stage including the committed-step gene *murA*, and 63% of the maturation-stage genes (Supplementary Fig. S20). Among the up-regulated genes, eight (*glmU, murA, murB, murC, murI, mrcA, dacA*, and *pbpG*,) have been experimentally identified as ethanol tolerance genes (Supplementary Tables S2 and S14). Furthermore, the effectiveness of this cell-wall reinforcement has been experimentally confirmed by over-expressing a *Lactobacillus plantarum murA* gene in *E. coli*^23^, and increased peptidoglycan synthesis provides physical protection against ethanol lytic effects^6^.

The proportion of unsaturated fatty acids, such as C18:1, in phospholipids is found to increase in response to ethanol^1^,^2^,^6^,^24^. The increased gene expression involved in fatty acid biosynthesis is given in Supplementary Results (Figs S27–S30). Such increased synthesis of unsaturated fatty acids will ameliorate the movement constraint on fatty acids imposed by ethanol inserted into membranes and aid restoring membrane proton potential^1^,^3^.

#### Improved intracellular environment during evolution

**Figure 7:**
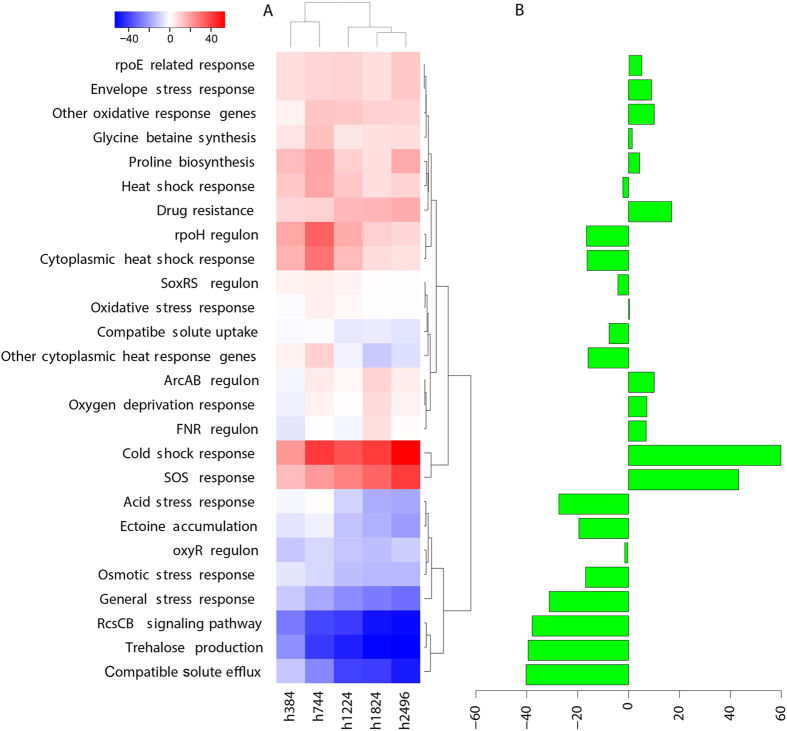
26 stress responses in six EA samples. (**A**) percentage of changes in mean expression level of each of the six strains to that of the parent strain in each response; (**B**) the difference (%) between time 0 and h2,496 in EA samples. The *x* axis represents the samples, and the *y* axis is for the proportion of differentially expressed genes.

Reduced stresses as an indicator for improved cellular milieu: Given the increased ATP generation and restored membrane potential, we expected that cells will have less stressful intracellular environment. A first indicator is the reduction in aberrant TU expression by EBP TFs. Specifically, no aberrantly expressed TUs of the repressive EBP TFs are found in the evolved strains; and only 42% of the TUs of two activating EBP TFs, NtrC and PhoP, are aberrantly expressed (Supplementary Fig. S21). Therefore, aberrant expression of TUs due to ethanol binding is significantly reduced compared to those in the NEA samples (Wilcoxon test, *p *<* *0.001). Since no mutations are directly involved, this reduction could be attributed to the strengthened cell wall and membranes, which may limit the invasion of ethanol molecules into the cells^25^. In addition, 12 stress-response pathways in the evolved strains see steady decrease compared to the parental strain, including the response levels of acid stress, general stress, osmotic stress, trehalose production, and the RcsCB signaling pathway (Fig. 7).

Mutations in *cspC, proQ* and *hns* may also contribute to the reduced stress responses. *cspC* encodes a ρ-independent anti-terminator, which has an IS5 insertion in strains E and F. Its knockout is shown to increase growth rate^20^, consistent with the reported growth gain due to function loss of this gene^26^. Mechanistically, mutated *cspC* decreases the stability of the *rpoS* mRNA, which in turn decreases the expression level of stress response gene *proP* (osmolyte:H^+^ symporter)^27^, giving rise to reduced levels of both the general and osmotic stress responses (Figs 7 and S22). *proQ* is an RNA chaperone that post-transcriptionally controls the expression level of *proP*^28^. A mutation (Leu91Gln) occurs in its critical protein region, contributing to the reduced expression of *proP. hns* is a global dual-regulator, and an IS5 insertion in the promoter regions in five of the six evolved strains (Supplementary Table S11) leads to its up-regulation in these strains (Supplementary Fig. S23A). Consequently, some *hns* regulon genes are differentially expressed (Supplementary Fig. S23B). Among the down-regulated genes, *gadW, nhaA* and *nhaR* are involved in acid-stress response, and *osmC* involved in osmotic stress response (Supplementary Fig. S23D), resulting in reduced responses to these two stress types.

#### Up-regulated stress response as effective counter resistance

Besides the above reduced stress responses, ten stress responses are increased to varying degrees. SOS response and cold shock response are among the most up-regulated (Fig. 7). These two processes have high positive correlations with DNA replication with correlation coefficients of 0.75 and 0.72, respectively. In contrast to the decreasing stress pathways as ‘passive’ indicators of improved cellular milleu, these two up-regulated responses may act as ‘active’ counter resistance to ethanol-induced stresses.

Ethanol induces lipid modification and stalling of gene expression^1^,^24^,^29^, analogous to the main effects of cold shock^24^,^30^. For the SOS response, 78% (21/27) genes are expressed (Supplementary Fig. S24A) and nine are consistently up-regulated during evolution (Supplementary Fig. S24B), including five key DNA repair genes *recN, uvrD, cho, dinG*, and *dinB*. The specific function of each differentially expressed gene is provided in Supplementary Table S18. Since the ROS level is low in the EA samples and therefore low DNA damage, SOS response may be active to induce error-prone mismatches, thus increasing the mutation rate for adaptation to ethanol^31^,^32^. In addition, most cold-shock protein (*csp*) genes are up-regulated with genes involved in DNA repair, transcription, and translation (Supplementary Fig. S25A). Among them, six genes: *cspA, deaS, hns, hscA, hscB* and *rbfA* show step-wise up-regulation (Supplementary Fig. S25B), with *hscA* being a known ethanol tolerance gene (Supplementary Table S14). The functions of the cold-shock response genes are provided in Supplementary Table S19. Two mutated genes are involved in this response: *hns* and *relA. hns*, a global dual-regulator, is a key organizing protein involved in chromosome structure (supercoiling) and shows step-wise up-regulation due to an IS5 insertion in five of the six EA strains (Supplementary Fig. S23A). Another mutated gene *relA* has three missense SNPs, each in one EA strain (C, E and F) and two of them in critical domains (Supplementary Table S11); this functional loss in *relA* is consistent with the observation that *relA* is down-regulated in all six EA strains. Furthermore, mutated *relA* has been shown to confer the biggest fitness gain among all identified mutations in strain F^20^. *RelA* is a synthase for the global regulatory molecules (p)ppGpp, which activates the expression of cold-shock proteins^33^. Therefore, mutated *relA* appears to synthesize fewer (p)ppGpp, causing lower expressions of the cold-shock proteins. We verified this with the expression level of ribosomal RNA genes, which is reduced by (p)ppGpp^34^. Therefore, the up-regulation of ribosomal biogenesis genes should be a result of mutated or down-regulated *relA*; indeed, this is what we observed in six EA strains (Supplementary Fig. S26). Therefore, both these reduced ‘passive’ and increased ‘active’ stress response both contributed to growth restoration in the EA strains.

#### Coordinated restoration of biological processes in the EA strains

**Figure 8:**
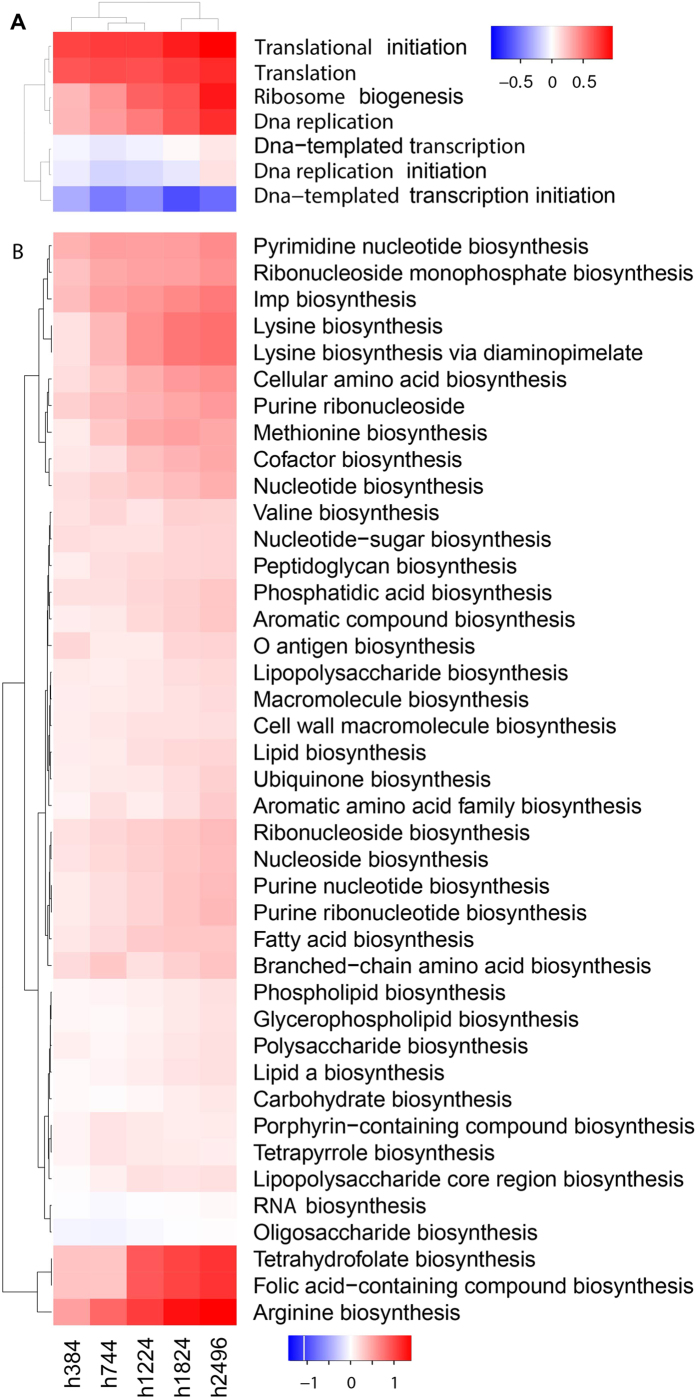
Time-dependent changes in transcription, translation, DNA replication and biosynthetic processes in six EA strain during evolution. (**A**) transcription, translation, DNA replication; and (**B**) biosynthetic processes.

To fully understand the adaptation process to ethanol-induced stresses, we compared differential expressions of genes involved in stress responses, transcription, translation, DNA replication, and biosynthetic processes during evolution, to detect time-dependent patterns, particularly monotonic patterns, at a systems level. First, most processes gradually recover or improve with multiple, not one, steps (time points) (Figs 7 and 8). Second, these improvements appear to proceed in a coordinated fashion with certain processes taking place prior to others. Fox example, translation and biosynthesis of amino acids and nucleotides recover first, followed by biosynthesis of macromolecules including peptidoglycan and fatty acids; and the last to recover is transcription and DNA replication (Fig. 8). Most stress responses show a steady decline or increase (e.g., glycine betaine biosynthesis and heat shock response). Third, there is a systems-level optimization of biological processes: essential pathways are enhanced (e.g., aerobic respiration) and non-essential pathways (e.g., PTS system) are repressed at certain points during the adaptive evolution. Last, an optimized system provides stronger tolerance against ethanol and improved growth in the presence of ethanol.

## Discussion

A key limiting factor in ethanol production is ethanol’s toxicity to the host cells. Despite extensive studies on this subject since 1970s, e.g., refs 2, 6, 35, 36, 37, new targets affected by ethanol continue to be identified, e.g., ref. 29. Therefore, a comprehensive systems-level understanding of ethanol toxicity is needed. By comparatively analyzing transcriptomic and genomic data of NEA vs. EA samples and known ethanol tolerance genes, we have proposed a model for how ethanol stresses the host cells and how stressed cells develop tolerance through evolution, both at a systems level. In our model, ethanol induces stresses at multiple levels of cellular activities, spanning a wide range of cellular processes. They include cell envelope integrity, multiple stress types, increased ROS production and reduced O_2_ level, compensated ATP production via three different mechanisms, macromolecule biosynthesis, and protein-ethanol binding and altered protein functions.

Our key findings are that ethanol disrupts the following processes: (i) weakening cell wall and cell membranes, leading to reduced cross-membrane proton gradient and hence reduced ATP production; (ii) induction of multiple stress types; (iii) increased ROS production due to stresses, leading to reduced intracellular O_2_ level and hence decreased aerobic respiration for ATP production; (iv) the reduced ATP production leading to a number of cellular-level changes such as reduced biosynthesis of macromolecules and therefore reduced cell proliferation, and increased fatty acid oxidation for energy production; and (v) altered functions of over 200 proteins including dozens of transcription factors, leading to extensive impact on many biological processes; and (vi) that these changes are functionally linked with each other, all stemming from the reduced cross-membrane proton gradient and increased intracellular ROS. We anticipate that this new understanding would guide more effective designing of better ethanol-tolerant strains.

We also proposed a model for how the evolved *E. coli* strains adapted to the ethanol-induced stresses and regained viability through selection of specific mutations, which enabled changes in certain basic behaviors of the cells, particularly in reducing ATP consumption for non-essential processes. The regained ATP sufficiency and biosynthesis of macromolecules put the cells on an evolutionary trajectory towards restoration. Our key findings are: (1) reduced non-essential energy consumption enabled by mutations allows the stressed cells to use ATP towards more essential activities for their survival, such as increased biosynthesis towards strengthened cell membrane, which in turn enables the cells to gradually regain their cross-membrane proton gradients and hence ATP production through respiration, leading to reduced overall stress level and hence ROS; (2) the reduced ROS production increases the intracellular O_2_ level for aerobic respiration, offering the most essential foundation for recovering the normal cellular functions; (3) the increased biosynthesis of macromolecules coupled with up-regulation of ethanol tolerance genes, allow for increased cell division; and (4) all these activities together improve the overall intracellular condition.

One defining characteristic of our model is that all processes affected by ethanol are interconnected through energy and stresses, in both the stressed and evolved cells. Thus, our model can serve as a unifying framework for integrating all ethanol targets identified so far. Previously identified impacts such as cell membrane proton gradient^4^ and ROS production^13^,^38^ can be viewed as the foundational changes; alterations in respiration (e.g., ArcAB regulon)^9^ and anaerobic fatty acid oxidation are the next-level responses; and the reduced translation and biosynthesis of macromolecules are the last and most comprehensive impact. The whole process is reverted in the evolved cells but starts with mutation-enabled reduction in non-essential ATP consumption, which gradually leads to improved biosynthesis, reduced stresses and improved ATP generation in an iterative manner, and finally increased DNA replication and growth. We have found two experimental supports for our model. For example, fortifying cell envelope by overexpressing genes, e.g., *murAG* for peptidoglycan biosynthesis and for unsaturated fatty biosynthesis^3^,^23^,^24^ have been successful in making ethanol tolerance bacteria. Ethanol-affected proteins (e.g., *rho, metJ*, and *rpsQ*) in transcription and translation are reinforced through mutations, so as to be more robust in the presence of ethanol^29^.

ROS production is an important finding in our study, which can be generated due to stresses as well as from damaged electron transport chain^39^. Another major finding is that ethanol can directly affect protein functions, a question long suspected but not confirmed at the physiological concentrations in bacteria^6^,^40^,^41^. A most ‘direct’ evidence in this regard comes from *rps,* which acquired beneficial mutations and reduced translational misreading in ethanol stressed cells^29^. We showed that ethanol alters protein functions by demonstrating that TFs can have their functions altered when bound with ethanol, hence impacting the functions at a pathway level. This may be the very reason why overexpressing protein chaperones GroESL increases ethanol tolerance in *E. coli*^42^.

Besides mutations and adaptive gene expression, microRNAs and non-coding RNAs have also been found to confer tolerance to stressors^43^,^44^,^45^,^46^. As most of microarray probes used in this study (Table 1) are not designed for these small RNAs, we cannot confirm their contribution. Future work is needed to study this issue.

Our qualitative model may need mechanistic validation with experimental studies, which could lead to a further improved quantitative model and better understanding about the fundamentals of ethanol stress, responses and adaptation. The merit of the study lies in a systems-level integration of a collection of observations about individual changes. Together, the model sheds new light on the complex issues about ethanol stresses and adaptation.

## Data and Methods

### *E. coli* transcriptomic data

Four sets of microarray gene-expression data, collected on three *E. coli* strains with ethanol treatment and control, are downloaded from the NCBI Gene Expression Omnibus^47^ and used in this study. The three strains are W3110, BW25113, and DH5α, which differ in coding regions of a few genes. For the collected data, these strains are grown in minimal (M9 or MOPS) media supplemented with glucose, and most are treated with 2.5%~5% (v/v) ethanol and one with a 15% ethanol shock for 15 min. Three of the four datasets are produced on Affymetrix microarrays (Santa Clara, CA, USA) and one on custom arrays, all covering at least 4,305 genes of *E. coli* K12 MG1655. Three datasets are for non-ethanol-adapted cells and one for ethanol-adapted cells. Table 1 summarizes the detailed information of these datasets. In the NEA samples, each treatment has three or eight replicates and the corresponding control has three or four replicates. In the EA samples, data are collected on six strains evolved in parallel from a common parent (GSE59050) at five time points: 384, 744, 1,224, 1,824, and 2,496 hours with ethanol treatment.

### Differential gene-expression analysis and functional enrichment

The three NEA datasets have a total of seven treatment-*versus-*control pairs (Table 1 and S1). The EA dataset has five evolved-*versus*-the-parental-strain pairs. Differential gene-expression analyses were conducted between each treatment or evolved sample and the matching control sample. For the NEA pairs, gene-expression data were analyzed using the R packages GEOquery^48^ and affy^49^. A differentially expressed gene (DEG) between each pair of samples is defined as having a fold change in expression >1.2 or <1/1.2 for up-regulation or down-regulation in Wilcoxon signed- rank test (*p* < 0.05), respectively. For the EA datasets, differential expression analyses were performed between the parental strain and each of the six evolved strains, each collected at five time points during the adaptive evolution in ethanol^20^ (Supplementary Table S1). For the EA samples, we used more stringent criteria for determining DEGs since there was only one control, i.e., the parental strain, *versus* six evolved strains: the six evolved strains must all have greater (or less) expression levels than the parent strain, and the mean difference is greater than 20% compared to the expression level in the parent strain.

A pathway enrichment analysis was conducted separately over the up- and down-regulated genes using DAVID^50^ against the Gene Ontology database^51^.

### Identification of ethanol-binding proteins

We predicted ethanol-binding proteins (EBPs) using a structure-based computational approach. The reference structure is taken from alcohol dehydrogenase (AdhE), the only known ethanol-binding enzyme in *E. coli*, which catalyzes the inter-conversion between ethanol and acetyl-CoA^9^,^52^. Specifically, we searched the protein structural data in the Protein Data Bank^53^ using keyword “alcohol dehydrogenase” in three enzyme classes: 1.1.1.1, 1.1.1.2 and 1.1.2.8, which gives rise to 130, 27 and 3 structures, respectively. Since all structures in the same class were highly similar, only one representative was chosen from each class: namely 2jhf, 4cvb and 1zk4 for ethanol binding analysis. We then downloaded the structural models for 3,784 *E. coli* proteins predicted by QUARK^54^, a state-of-the-art prediction program. These models were structurally aligned to 2jhf, 4cvv, and 1zk4 using SP-align^55^ for identification of possible binding sites with ethanol. It is noteworthy that structural alignment for binding pocket identification by tools like SP-align has been shown to be powerful enough for identifying proteins with the same molecular function^55^,^56^,^57^,^58^. 213 *E. coli* proteins are found to have similar binding pockets with the three alcohol dehydrogenase proteins with SP-score >0.5, a widely used threshold for identifying proteins with the same structural fold. Functional information of these proteins, including proteins that interact with, are retrieved from the UniProt database^59^.

### Altered gene regulation by ethanol-bound transcription factors

Among the 213 predicted EBPs, 23 are transcription factors (TFs). So we analyzed the expression profiles of the transcription units (TUs) regulated by each of these TFs as defined in regulonDB^60^, to determine whether their regulatory functions are altered by ethanol binding. We used TUs that are regulated by EBP TFs only. To quantify aberrant expressions for each such TU, we first calculate the differential expression of the TF and genes in each TU between control and ethanol treated samples. We then divide the TUs into two categories: activated or repressed based on the direction of the regulation. For an activated TU, it is considered as having aberrant expression if (1) the TF is not differentially expressed but a TU is down-regulated, or (2) the TF is up-regulated but the TU is not. For a repressed TU, it is considered as aberrantly expressed if (1) the TF is not differentially expressed but a TU is up-regulated, or (2) the TF is up-regulated and the TU is also up-regulated. The level of aberrantly expressed TUs is defined as the percentage of the all the TUs of TF’s regulon.

### Ethanol tolerance genes and stress response pathways

Numerous genes have been found to increase ethanol tolerance in *E. coli* when overexpressed or knocked out^7^,^42^,^61^. We found these genes (Supplementary Table S2) through a systematic literature search using keywords ‘ethanol tolerance’ and ‘*E. coli*’ in Web of Science and PubMed. We also collected 26 stress-response genes (Supplementary Table S3) as identified by Storz and Hengge^62^ and curated by Goodarzi *et al*.^9^, respectively.

## Additional Information

**How to cite this article**: Cao, H. *et al*. Systems-level understanding of ethanol-induced stresses and adaptation in *E. coli.* *Sci. Rep.* **7**, 44150; doi: 10.1038/srep44150 (2017).

**Publisher's note:** Springer Nature remains neutral with regard to jurisdictional claims in published maps and institutional affiliations.

## Supplementary Material

Supplementary Information

Supplementary Dataset 1

Supplementary Dataset 2

Supplementary Dataset 3

Supplementary Dataset 4

Supplementary Dataset 5

## Figures and Tables

**Table 1:** Four microarray datasets containing seven control-treatment comparisons in response to ethanol in two *E. coli* strains that are not evolved, and one strain that is evolved for 2,496 h with six replicates in the presence of ethanol.

Accession No.	Sample name	Ethanol (v/v)	Replicate	*E. coli* K12 strain	Microarray platform	Medium
GSE3665	GSE3665A^b^	0 vs. 2.5%	6 (3 + 3)	DH5α	GPL3154	M9 with 5 g/L D-glucose and 1 mM thiamine
	GSE3665B^b^	0 vs. 5%	12 (8 + 4)	DH5α	GPL3154	M9 with 5 g/L D-glucose and 1 mM thiamine
GSE13444	GSE13444	3%	12 (8 + 4)	BW25113	GPL5113	MOPS with 0.2% glucose
GSE17526	GSE17526A^b^	15% (15 min)	6 (3 + 3)	BW25113	GPL3154	M9 + 4 g/L glucose
	GSE17526B^b^	15% (15 min)	6 (3 + 3)	BW25113	GPL3154	M9 + 4 g/L glucose
	GSE17526C^b^	15% (15 min)	6 (3 + 3)	BW25113	GPL3154	M9 + 4 g/L glucose
	GSE17526D^b^	15% (15 min)	6 (3 + 3)	BW25113	GPL3154	M9 + 4 g/L glucose
GSE59050^a^	Parent/control	5%	6	W3310	GPL13336	M9 + 4 g/L glucose
	h384	5%	6	W3310	GPL13336	M9 + 4 g/L glucose
	h744	5%	6	W3310	GPL13336	M9 + 4 g/L glucose
	h1,224	5%	6	W3310	GPL13336	M9 + 4 g/L glucose
	h1,824	5%	6	W3310	GPL13336	M9 + 4 g/L glucose
	h2,496	5%	6	W3310	GPL13336	M9 + 4 g/L glucose

^a^This data set contains parent (non-evolved) strain and evolved strains for 2,496 h in the presence of ethanol.

^b^These samples are used in correlation analyses with different biological processes.
