# Supplementary material for: Systems-level understanding of ethanol-induced stresses and adaptation in E. coli
Source: Sci Rep. 2017 Mar 16;7:44150. doi: 10.1038/srep44150 (PMC5353561; doi:10.1038/srep44150)

**Systems-level understanding of ethanol-induced stresses and adaptation in *E. coli***

Huansheng Cao^1,2,ǂ^, Wei Du^1,3,ǂ^, Yuedong Yang^4^, Yu Shang^1,3^, Gaoyang Li^1,3^, Yaoqi Zhou^4^, Qin Ma^5^ and Ying Xu^1,2,3,a^

^1^Computational Systems Biology Laboratory, Department of Biochemistry and Molecular Biology, and Institute of Bioinformatics, the University of Georgia, Athens, GA 30602, USA

^2^BioEnergy Science Center, Oak Ridge National Laboratory, Oak Ridge, TN 37831, USA

^3^College of Computer Science and Technology, Jilin University, Changchun, 130012, China

^4^Institute for Glycomics and School of Information and Communication Technology, Griffith University, Parklands Dr., Southport, QLD 4222, Australia

^5^Department of Agronomy, Horticulture and Plant Science, South Dakota State University, Brookings, SD 57007, USA; and BioSNTR, Brookings, SD, 57007, USA

^ǂ^These authors contributed equally

^a^ Corresponding author: xyn@uga.edu

**Supplementary Information:**

Supplementary Results

Supplementary Tables S1-S19

Supplementary Figures S1-S29

# Supplementary results

## **Biosynthesis of fatty acids**

We observed upregulated biosynthesis of fatty acids in all six EA strains. First, the repressor (*fadR*) of fatty acid oxidation is upregulated so as to repress oxidation in the EA samples; also upregulated is the long-chain fatty acid transporter (*fadL*) (Supplementary Fig. S26A). Moreover, these two genes are consistently upregulated over the five time point of evolution (Supplementary Fig. S26B). Next, most genes in fatty acid biosynthesis are upregulated (Supplementary Fig. S27). Of these genes, *accABCD* product catalyzes the biosynthesis of the pathway donor: malonyl-CoA. *fabA* and *fabZ* products both catalyze the unsaturation (double bonds) of fatty acids. *accA*, *accC* and *fabZ* are all consistently upregulated during evolution (Supplementary Fig. S28). Then fatty acids are incorporated into CDP-diacylglycerol biosynthesis with genes shown in Supplementary Fig. S29A; one gene (*plsC*) is consistently upregulated during evolution (Supplementary Fig. S29B).

# **Supplementary Table Legends**

**Supplementary Table S1 The transcriptional and genomic datasets collected for non-ethanol-adapted and ethanol-adapted *E. coli*** K12 strains.

| GSE13444 | Pair A | Control | GSM336463 | Ecoli_wildtype_calibration_Sample1_TechRep1 |
| --- | --- | --- | --- | --- |
|  |  |  | GSM336464 | Ecoli_wildtype_calibration_Sample1_TechRep2 |
|  |  |  | GSM336481 | Ecoli_wildtype_calibration_Sample2_TechRep2 |
|  |  |  | GSM336571 | Ecoli_wildtype_calibration_Sample2_TechRep1 |
|  |  | Treatment | GSM336488 | Ecoli_wildtype_3%_ethanol_Sample1_TechRep1 |
|  |  |  | GSM336489 | Ecoli_wildtype_3%_ethanol_Sample1_TechRep2 |
|  |  |  | GSM336493 | Ecoli_wildtype_3%_ethanol_Sample2_TechRep1 |
|  |  |  | GSM336494 | Ecoli_wildtype_3%_ethanol_Sample2_TechRep2 |
|  |  |  | GSM336497 | Ecoli_wildtype_3%_ethanol_Sample3_TechRep1 |
|  |  |  | GSM336498 | Ecoli_wildtype_3%_ethanol_Sample3_TechRep2 |
|  |  |  | GSM336505 | Ecoli_wildtype_3%_ethanol_Sample4_TechRep1 |
|  |  |  | GSM336506 | Ecoli_wildtype_3%_ethanol_Sample4_TechRep2 |
| GSE17526 | Pair A | Control | GSM436889 | BW25113 log, biological rep1 |
|  |  |  | GSM436890 | BW25113 log, biological rep2 |
|  |  |  | GSM436891 | BW25113 log, biological rep3 |
|  |  | Treatment | GSM436901 | BW25113 log ethanol shock, biological rep1 |
|  |  |  | GSM436902 | BW25113 log ethanol shock, biological rep2 |
|  |  |  | GSM436903 | BW25113 log ethanol shock, biological rep3 |
|  | Pair B | Control | GSM436892 | BW25113 stationary, biological rep1 |
|  |  |  | GSM436893 | BW25113 stationary, biological rep2 |
|  |  |  | GSM436894 | BW25113 stationary, biological rep3 |
|  |  | Treatment | GSM436904 | BW25113 stationary ethanol shock, biological rep1 |
|  |  |  | GSM436905 | BW25113 stationary ethanol shock, biological rep2 |
|  |  |  | GSM436906 | BW25113 stationary ethanol shock, biological rep3 |
|  | Pair C | Control | GSM436883 | BW25113 (rpoS-) log, biological rep1 |
|  |  |  | GSM436884 | BW25113 (rpoS-) log, biological rep2 |
|  |  |  | GSM436885 | BW25113 (rpoS-) log, biological rep3 |
|  |  | Treatment | GSM436895 | BW25113 (rpoS-) log ethanol shock, biological rep1 |
|  |  |  | GSM436896 | BW25113 (rpoS-) log ethanol shock, biological rep2 |
|  |  |  | GSM436897 | BW25113 (rpoS-) log ethanol shock, biological rep3 |
|  | Pair D | Control | GSM436886 | BW25113 (rpoS-) stationary, biological rep1 |
|  |  |  | GSM436887 | BW25113 (rpoS-) stationary, biological rep2 |
|  |  |  | GSM436888 | BW25113 (rpoS-) stationary, biological rep3 |
|  |  | Treatment | GSM436898 | BW25113 (rpoS-) stationary ethanol shock, biological rep1 |
|  |  |  | GSM436899 | BW25113 (rpoS-) stationary ethanol shock, biological rep2 |
|  |  |  | GSM436900 | BW25113 (rpoS-) stationary ethanol shock, biological rep3 |
| GSE3665 | PAIR A | Control | GSM85143 | wild type, 0 g/L EtOH, replicate 1 |
|  |  |  | GSM85144 | wild type, 0 g/L EtOH, replicate 2 |
|  |  |  | GSM85145 | wild type, 0 g/L EtOH, replicate 3 |
|  |  | Treatment | GSM85160 | wild type, 20 g/L EtOH, replicate 1 |
|  |  |  | GSM85161 | wild type, 20 g/L EtOH, replicate 2 |
|  |  |  | GSM85162 | wild type, 20 g/L EtOH, replicate 3 |
|  | PAIR B | Control | GSM85146 | round 1, 0 g/L EtOH, replicate 1 |
|  |  |  | GSM85147 | round 1, 0 g/L EtOH, replicate 2 |
|  |  |  | GSM85148 | round 1, 0 g/L EtOH, replicate 3 |
|  |  |  | GSM85149 | round 2, 0 g/L EtOH, replicate 1 |
|  |  |  | GSM85150 | round 2, 0 g/L EtOH, replicate 2 |
|  |  |  | GSM85151 | round 2, 0 g/L EtOH, replicate 3 |
|  |  |  | GSM85152 | round 3, 0 g/L EtOH, replicate 1 |
|  |  |  | GSM85153 | round 3, 0 g/L EtOH, replicate 2 |
|  |  | Treatment | GSM85154 | round 3, 20 g/L EtOH, replicate 1 |
|  |  |  | GSM85155 | round 3, 20 g/L EtOH, replicate 2 |
|  |  |  | GSM85156 | round 3, 20 g/L EtOH, replicate 3 |
|  |  |  | GSM85157 | round 3, 40 g/L EtOH, replicate 1 |
|  |  |  | GSM85158 | round 3, 40 g/L EtOH, replicate 2 |
|  |  |  | GSM85159 | round 3, 40 g/L EtOH, replicate 3 |
| GSE59050 |  | Control (parent strain) | GSM1424998 | Parent strain of experimental evolution |
|  |  | Evolution time point 1 | GSM1424999 | Evolved strain A, time point 1 |
|  |  |  | GSM1425004 | Evolved strain B, time point 1 |
|  |  |  | GSM1425009 | Evolved strain C, time point 1 |
|  |  |  | GSM1425014 | Evolved strain D, time point 1 |
|  |  |  | GSM1425019 | Evolved strain E, time point 1 |
|  |  |  | GSM1425024 | Evolved strain F, time point 1 |
|  |  | Evolution time point 2 | GSM1425000 | Evolved strain A, time point 2 |
|  |  |  | GSM1425005 | Evolved strain B, time point 2 |
|  |  |  | GSM1425010 | Evolved strain C, time point 2 |
|  |  |  | GSM1425015 | Evolved strain D, time point 2 |
|  |  |  | GSM1425020 | Evolved strain E, time point 2 |
|  |  |  | GSM1425025 | Evolved strain F, time point 2 |
|  |  | Evolution time point 3 | GSM1425001 | Evolved strain A, time point 3 |
|  |  |  | GSM1425006 | Evolved strain B, time point 3 |
|  |  |  | GSM1425011 | Evolved strain C, time point 3 |
|  |  |  | GSM1425016 | Evolved strain D, time point 3 |
|  |  |  | GSM1425021 | Evolved strain E, time point 3 |
|  |  |  | GSM1425026 | Evolved strain F, time point 3 |
|  |  | Evolution time point 4 | GSM1425002 | Evolved strain A, time point 4 |
|  |  |  | GSM1425007 | Evolved strain B, time point 4 |
|  |  |  | GSM1425012 | Evolved strain C, time point 4 |
|  |  |  | GSM1425017 | Evolved strain D, time point 4 |
|  |  |  | GSM1425022 | Evolved strain E, time point 4 |
|  |  |  | GSM1425027 | Evolved strain F, time point 4 |
|  |  | Evolution time point 5 | GSM1425003 | Evolved strain A, time point 5 |
|  |  |  | GSM1425008 | Evolved strain B, time point 5 |
|  |  |  | GSM1425013 | Evolved strain C, time point 5 |
|  |  |  | GSM1425018 | Evolved strain D, time point 5 |
|  |  |  | GSM1425023 | Evolved strain E, time point 5 |
|  |  |  | GSM1425028 | Evolved strain F, time point 5 |

**Supplementary Table S2. The ethanol tolerance genes that are identified experimentally via overexpression or knocking out.**

(This Table is provided in an excel spreadsheet due to its big size.)

**Supplementary Table S3. The stress response pathways in *E. coli*.**

(This Table is provided in an excel spreadsheet due to its big size.)

**Supplementary Table S4. The correlation of DNA replication, energy taxis, and biosynthetic processes with ethanol concentration in the NEA samples of *E. coli***.

| GO term | Ethanol |
| --- | --- |
| DNA replication | -0.45 |
| Energy taxis | 0.42 |
| tetrapyrrole biosynthesis | -0.51 |
| porphyrin-containing compound biosynthesis | -0.50 |
| cell wall macromolecule biosynthesis | -0.46 |
| ubiquinone biosynthesis | -0.44 |
| cofactor biosynthesis | -0.32 |
| folic acid-containing compound biosynthesis | -0.27 |
| lysine biosynthesis process via diaminopimelate | -0.26 |
| lysine biosynthesis | -0.26 |
| regulation of RNA biosynthesis | -0.26 |
| phospholipid biosynthesis | -0.25 |

**Supplementary Table S5. The correlation between biosynthetic processes and DNA replication in the presence of ethanol in the samples of *E. coli***.

| GO Terms | DNA replication |
| --- | --- |
| ubiquinone biosynthetic process | 0.584754802 |
| tetrapyrrole biosynthetic process | 0.538460837 |
| porphyrin-containing compound biosynthetic process | 0.533433182 |
| peptidoglycan biosynthetic process | 0.441516611 |
| cell wall macromolecule biosynthetic process | 0.440352648 |
| glycerophospholipid biosynthetic process | 0.408525218 |
| cofactor biosynthetic process | 0.402238157 |
| phospholipid biosynthetic process | 0.401112745 |
| O antigen biosynthetic process | 0.337943 |
| lipid A biosynthetic process | 0.333104136 |
| tetrahydrofolate biosynthetic process | 0.331443984 |
| folic acid-containing compound biosynthetic process | 0.330838596 |
| nucleotide-sugar biosynthetic process | 0.306313824 |
| branched-chain amino acid biosynthetic process | 0.296349025 |
| phosphatidic acid biosynthetic process | 0.295693152 |
| nucleoside biosynthetic process | 0.264047388 |
| aromatic compound biosynthetic process | 0.255482437 |
| ribonucleoside biosynthetic process | 0.252975323 |
| valine biosynthetic process | 0.250115749 |
| lysine biosynthetic process via diaminopimelate | 0.250107929 |
| lysine biosynthetic process | 0.250107929 |
| nucleotide biosynthetic process | 0.246119178 |
| lipid biosynthetic process | 0.241940273 |
| purine nucleotide biosynthetic process | 0.241142774 |

**Supplementary Table S6. Ethanol-binding proteins computationally identified based on the structure of alcohol dehydrogenase (AdhE) in *E. coli*.**

(This Table is provided in an excel spreadsheet due to its big size.)

**Supplementary Table S**7. The functional enrichment of ethanol-binding proteins in terms of GO biological function terms.

| Term | Count | % | PValue | Fold Enrichment | Bonferroni | Benjamini | FDR |
| --- | --- | --- | --- | --- | --- | --- | --- |
| GO:0055114~oxidation reduction | 7.20E+01 | 3.50E+01 | 1.50E-22 | 3.45E+00 | 6.34E-20 | 6.34E-20 | 2.10E-19 |
| GO:0016052~carbohydrate catabolic process | 3.20E+01 | 1.55E+01 | 1.70E-13 | 5.03E+00 | 7.19E-11 | 3.59E-11 | 2.38E-10 |
| GO:0034637~cellular carbohydrate biosynthetic process | 2.60E+01 | 1.26E+01 | 1.99E-11 | 5.28E+00 | 8.42E-09 | 2.81E-09 | 2.79E-08 |
| GO:0046377~colanic acid metabolic process | 9.00E+00 | 4.37E+00 | 2.54E-10 | 3.14E+01 | 1.08E-07 | 2.70E-08 | 3.58E-07 |
| GO:0009242~colanic acid biosynthetic process | 9.00E+00 | 4.37E+00 | 2.54E-10 | 3.14E+01 | 1.08E-07 | 2.70E-08 | 3.58E-07 |
| GO:0016051~carbohydrate biosynthetic process | 2.60E+01 | 1.26E+01 | 3.60E-09 | 4.12E+00 | 1.53E-06 | 3.05E-07 | 5.06E-06 |
| GO:0033692~cellular polysaccharide biosynthetic process | 2.10E+01 | 1.02E+01 | 5.98E-09 | 5.05E+00 | 2.54E-06 | 4.23E-07 | 8.41E-06 |
| GO:0044264~cellular polysaccharide metabolic process | 2.10E+01 | 1.02E+01 | 1.57E-08 | 4.77E+00 | 6.66E-06 | 9.52E-07 | 2.21E-05 |
| GO:0008610~lipid biosynthetic process | 2.20E+01 | 1.07E+01 | 3.65E-08 | 4.32E+00 | 1.55E-05 | 1.93E-06 | 5.13E-05 |
| GO:0000271~polysaccharide biosynthetic process | 2.20E+01 | 1.07E+01 | 1.43E-07 | 3.98E+00 | 6.08E-05 | 6.76E-06 | 2.02E-04 |
| GO:0006733~oxidoreduction coenzyme metabolic process | 1.20E+01 | 5.83E+00 | 4.10E-07 | 7.77E+00 | 1.74E-04 | 1.74E-05 | 5.77E-04 |
| GO:0009103~lipopolysaccharide biosynthetic process | 1.60E+01 | 7.77E+00 | 8.09E-07 | 4.96E+00 | 3.43E-04 | 3.12E-05 | 1.14E-03 |
| GO:0008653~lipopolysaccharide metabolic process | 1.60E+01 | 7.77E+00 | 1.15E-06 | 4.83E+00 | 4.88E-04 | 4.07E-05 | 1.62E-03 |
| GO:0046496~nicotinamide nucleotide metabolic process | 1.00E+01 | 4.85E+00 | 2.28E-06 | 8.65E+00 | 9.67E-04 | 7.44E-05 | 3.21E-03 |
| GO:0009820~alkaloid metabolic process | 1.00E+01 | 4.85E+00 | 2.28E-06 | 8.65E+00 | 9.67E-04 | 7.44E-05 | 3.21E-03 |
| GO:0006769~nicotinamide metabolic process | 1.00E+01 | 4.85E+00 | 2.28E-06 | 8.65E+00 | 9.67E-04 | 7.44E-05 | 3.21E-03 |
| GO:0043603~cellular amide metabolic process | 1.20E+01 | 5.83E+00 | 2.75E-06 | 6.41E+00 | 1.16E-03 | 8.32E-05 | 3.86E-03 |
| GO:0019362~pyridine nucleotide metabolic process | 1.00E+01 | 4.85E+00 | 3.27E-06 | 8.28E+00 | 1.39E-03 | 9.24E-05 | 4.60E-03 |
| GO:0009451~RNA modification | 1.20E+01 | 5.83E+00 | 4.37E-06 | 6.11E+00 | 1.85E-03 | 1.16E-04 | 6.15E-03 |
| GO:0009225~nucleotide-sugar metabolic process | 8.00E+00 | 3.88E+00 | 4.69E-06 | 1.18E+01 | 1.99E-03 | 1.17E-04 | 6.59E-03 |
| GO:0009110~vitamin biosynthetic process | 1.40E+01 | 6.80E+00 | 5.95E-06 | 4.89E+00 | 2.52E-03 | 1.40E-04 | 8.36E-03 |
| GO:0051186~cofactor metabolic process | 2.20E+01 | 1.07E+01 | 6.35E-06 | 3.14E+00 | 2.69E-03 | 1.42E-04 | 8.93E-03 |
| GO:0042364~water-soluble vitamin biosynthetic process | 1.30E+01 | 6.31E+00 | 7.66E-06 | 5.21E+00 | 3.24E-03 | 1.62E-04 | 1.08E-02 |
| GO:0005976~polysaccharide metabolic process | 2.20E+01 | 1.07E+01 | 1.17E-05 | 3.02E+00 | 4.94E-03 | 2.36E-04 | 1.64E-02 |
| GO:0019748~secondary metabolic process | 1.10E+01 | 5.34E+00 | 1.57E-05 | 5.97E+00 | 6.64E-03 | 3.03E-04 | 2.21E-02 |
| GO:0019520~aldonic acid metabolic process | 5.00E+00 | 2.43E+00 | 1.71E-05 | 3.05E+01 | 7.23E-03 | 3.15E-04 | 2.41E-02 |
| GO:0019521~D-gluconate metabolic process | 5.00E+00 | 2.43E+00 | 1.71E-05 | 3.05E+01 | 7.23E-03 | 3.15E-04 | 2.41E-02 |
| GO:0006766~vitamin metabolic process | 1.40E+01 | 6.80E+00 | 1.88E-05 | 4.38E+00 | 7.96E-03 | 3.33E-04 | 2.65E-02 |
| GO:0000154~rRNA modification | 7.00E+00 | 3.40E+00 | 1.95E-05 | 1.24E+01 | 8.23E-03 | 3.30E-04 | 2.74E-02 |
| GO:0006732~coenzyme metabolic process | 1.70E+01 | 8.25E+00 | 2.39E-05 | 3.55E+00 | 1.01E-02 | 3.89E-04 | 3.35E-02 |
| GO:0006767~water-soluble vitamin metabolic process | 1.30E+01 | 6.31E+00 | 2.62E-05 | 4.60E+00 | 1.11E-02 | 4.12E-04 | 3.69E-02 |

**Supplementary Table S**8. The functional enrichment of aberrantly expressed transcription units regulated EBP TFs in terms of GO biological function terms.

| Term | Count | % | PValue | Fold Enrichment | Bonferroni | Benjamini | FDR |
| --- | --- | --- | --- | --- | --- | --- | --- |
| GO:0009309~amine biosynthetic process | 8 | 17.3913 | 1.72E-04 | 6.365333 | 0.032263 | 0.032263 | 0.213358 |
| GO:0043648~dicarboxylic acid metabolic process | 5 | 10.86957 | 3.37E-04 | 14.46667 | 0.062435 | 0.03172 | 0.418992 |
| GO:0005977~glycogen metabolic process | 3 | 6.521739 | 6.02E-04 | 79.56667 | 0.108729 | 0.037642 | 0.746866 |
| GO:0006112~energy reserve metabolic process | 3 | 6.521739 | 6.02E-04 | 79.56667 | 0.108729 | 0.037642 | 0.746866 |
| GO:0006970~response to osmotic stress | 3 | 6.521739 | 0.00147 | 51.15 | 0.244898 | 0.067817 | 1.812825 |
| GO:0044271~nitrogen compound biosynthetic process | 9 | 19.56522 | 0.001923 | 3.691237 | 0.307679 | 0.070902 | 2.36634 |
| GO:0015837~amine transport | 5 | 10.86957 | 0.003127 | 7.930233 | 0.4502 | 0.094891 | 3.821039 |
| GO:0006073~cellular glucan metabolic process | 3 | 6.521739 | 0.004631 | 28.644 | 0.587949 | 0.118965 | 5.610743 |
| GO:0008652~cellular amino acid biosynthetic process | 6 | 13.04348 | 0.004793 | 5.208 | 0.600515 | 0.108364 | 5.800938 |
| GO:0044042~glucan metabolic process | 3 | 6.521739 | 0.00519 | 27.02264 | 0.629843 | 0.104547 | 6.26757 |
| GO:0016051~carbohydrate biosynthetic process | 6 | 13.04348 | 0.007741 | 4.642464 | 0.773348 | 0.137943 | 9.214602 |
| GO:0015847~putrescine transport | 2 | 4.347826 | 0.008115 | 238.7 | 0.789092 | 0.131932 | 9.6393 |
| GO:0006571~tyrosine biosynthetic process | 2 | 4.347826 | 0.010134 | 190.96 | 0.857081 | 0.149663 | 11.90062 |
| GO:0006570~tyrosine metabolic process | 2 | 4.347826 | 0.010134 | 190.96 | 0.857081 | 0.149663 | 11.90062 |
| GO:0009073~aromatic amino acid family biosynthetic process | 3 | 6.521739 | 0.010425 | 18.84474 | 0.864884 | 0.142703 | 12.22216 |
| GO:0046417~chorismate metabolic process | 3 | 6.521739 | 0.010689 | 18.6 | 0.871608 | 0.136377 | 12.51352 |
| GO:0009072~aromatic amino acid family metabolic process | 3 | 6.521739 | 0.011777 | 17.68148 | 0.895932 | 0.140021 | 13.70212 |
| GO:0046394~carboxylic acid biosynthetic process | 6 | 13.04348 | 0.011849 | 4.181606 | 0.897381 | 0.132635 | 13.78089 |
| GO:0016053~organic acid biosynthetic process | 6 | 13.04348 | 0.012059 | 4.163372 | 0.901462 | 0.12743 | 14.00847 |
| GO:0009094~L-phenylalanine biosynthetic process | 2 | 4.347826 | 0.01416 | 136.4 | 0.934377 | 0.140431 | 16.2553 |
| GO:0006558~L-phenylalanine metabolic process | 2 | 4.347826 | 0.01416 | 136.4 | 0.934377 | 0.140431 | 16.2553 |
| GO:0009095~aromatic amino acid family biosynthetic process, prephenate pathway | 2 | 4.347826 | 0.016167 | 119.35 | 0.955535 | 0.151126 | 18.35142 |
| GO:0009628~response to abiotic stimulus | 3 | 6.521739 | 0.016909 | 14.61429 | 0.961503 | 0.150289 | 19.11432 |
| GO:0009084~glutamine family amino acid biosynthetic process | 3 | 6.521739 | 0.022824 | 12.45391 | 0.987845 | 0.189415 | 24.96476 |
| GO:0000271~polysaccharide biosynthetic process | 5 | 10.86957 | 0.023233 | 4.42037 | 0.988777 | 0.184606 | 25.354 |
| GO:0005978~glycogen biosynthetic process | 2 | 4.347826 | 0.024154 | 79.56667 | 0.990629 | 0.183762 | 26.22536 |
| GO:0006006~glucose metabolic process | 4 | 8.695652 | 0.034024 | 5.440456 | 0.998655 | 0.240797 | 34.98837 |
| GO:0005996~monosaccharide metabolic process | 5 | 10.86957 | 0.038309 | 3.776899 | 0.999425 | 0.258021 | 38.48628 |
| GO:0015846~polyamine transport | 2 | 4.347826 | 0.039942 | 47.74 | 0.999584 | 0.258766 | 39.77284 |
| GO:0006796~phosphate metabolic process | 4 | 8.695652 | 0.052517 | 4.568421 | 0.999966 | 0.317247 | 48.88353 |
| GO:0005976~polysaccharide metabolic process | 5 | 10.86957 | 0.055152 | 3.352528 | 0.99998 | 0.320899 | 50.6237 |
| GO:0044264~cellular polysaccharide metabolic process | 4 | 8.695652 | 0.056257 | 4.44093 | 0.999984 | 0.317061 | 51.33738 |
| GO:0019318~hexose metabolic process | 4 | 8.695652 | 0.06177 | 4.272036 | 0.999995 | 0.333648 | 54.75759 |
| GO:0006793~phosphorus metabolic process | 4 | 8.695652 | 0.063103 | 4.234146 | 0.999996 | 0.330754 | 55.55084 |
| GO:0019438~aromatic compound biosynthetic process | 3 | 6.521739 | 0.065041 | 6.986341 | 0.999997 | 0.330628 | 56.68126 |
| GO:0006596~polyamine biosynthetic process | 2 | 4.347826 | 0.068877 | 27.28 | 0.999999 | 0.33837 | 58.84115 |
| GO:0034637~cellular carbohydrate biosynthetic process | 4 | 8.695652 | 0.07353 | 3.970062 | 1 | 0.348865 | 61.32746 |
| GO:0009089~lysine biosynthetic process via diaminopimelate | 2 | 4.347826 | 0.082089 | 22.73333 | 1 | 0.373391 | 65.54449 |
| GO:0046451~diaminopimelate metabolic process | 2 | 4.347826 | 0.082089 | 22.73333 | 1 | 0.373391 | 65.54449 |
| GO:0006553~lysine metabolic process | 2 | 4.347826 | 0.082089 | 22.73333 | 1 | 0.373391 | 65.54449 |
| GO:0009085~lysine biosynthetic process | 2 | 4.347826 | 0.082089 | 22.73333 | 1 | 0.373391 | 65.54449 |
| GO:0009250~glucan biosynthetic process | 2 | 4.347826 | 0.085831 | 21.7 | 1 | 0.37881 | 67.25129 |
| GO:0006595~polyamine metabolic process | 2 | 4.347826 | 0.098809 | 18.72157 | 1 | 0.415537 | 72.58755 |
| GO:0009064~glutamine family amino acid metabolic process | 3 | 6.521739 | 0.099087 | 5.466412 | 1 | 0.408136 | 72.69251 |

**Supplementary Table S**9. Correlations of peptidoglycan biosynthesis with ethanol concentration and other biosynthetic processes in the NEA samples.

| GO Terms | Ethanol | Peptidoglycan |
| --- | --- | --- |
| tetrapyrrole biosynthesis | -0.507092553 | 0.579284113 |
| porphyrin-containing compound biosynthesis | -0.49913816 | 0.572919231 |
| cell wall macromolecule biosynthesis | -0.456966163 | 0.469227515 |
| ubiquinone biosynthesis | -0.443987702 | 0.612504239 |
| cofactor biosynthesis | -0.320532577 | 0.405318093 |
| folic acid-containing compound biosynthesis | -0.272228634 | 0.316509795 |
| lysine biosynthesis process via diaminopimelate | -0.264110705 | 0.194002151 |
| lysine biosynthesis | -0.264110705 | 0.194002151 |
| regulation of RNA biosynthesis | -0.256697699 | 0.212176278 |
| phospholipid biosynthesis | -0.2500248 | 0.39447932 |

**Supplementary Table S10. Enriched GO biological processes by differentially expressed genes in the NEA (and EA) samples. The ‘up’ and ‘down’ in the first row of the table represent the number of processes that are up-regulated or down-regulated in both NEA and EA samples, respectively.**

| Term | Count | % | PValue | Fold Enrichment | Bonferroni | Benjamini | FDR |
| --- | --- | --- | --- | --- | --- | --- | --- |
| GO:0009309~amine biosynthetic process | 8 | 1.74E+01 | 1.72E-04 | 6.37E+00 | 3.23E-02 | 3.23E-02 | 2.13E-01 |
| GO:0043648~dicarboxylic acid metabolic process | 5 | 1.09E+01 | 3.37E-04 | 1.45E+01 | 6.24E-02 | 3.17E-02 | 4.19E-01 |
| GO:0005977~glycogen metabolic process | 3 | 6.52E+00 | 6.02E-04 | 7.96E+01 | 1.09E-01 | 3.76E-02 | 7.47E-01 |
| GO:0006112~energy reserve metabolic process | 3 | 6.52E+00 | 6.02E-04 | 7.96E+01 | 1.09E-01 | 3.76E-02 | 7.47E-01 |
| GO:0006970~response to osmotic stress | 3 | 6.52E+00 | 1.47E-03 | 5.12E+01 | 2.45E-01 | 6.78E-02 | 1.81E+00 |
| GO:0044271~nitrogen compound biosynthetic process | 9 | 1.96E+01 | 1.92E-03 | 3.69E+00 | 3.08E-01 | 7.09E-02 | 2.37E+00 |
| GO:0015837~amine transport | 5 | 1.09E+01 | 3.13E-03 | 7.93E+00 | 4.50E-01 | 9.49E-02 | 3.82E+00 |
| GO:0006073~cellular glucan metabolic process | 3 | 6.52E+00 | 4.63E-03 | 2.86E+01 | 5.88E-01 | 1.19E-01 | 5.61E+00 |
| GO:0008652~cellular amino acid biosynthetic process | 6 | 1.30E+01 | 4.79E-03 | 5.21E+00 | 6.01E-01 | 1.08E-01 | 5.80E+00 |
| GO:0044042~glucan metabolic process | 3 | 6.52E+00 | 5.19E-03 | 2.70E+01 | 6.30E-01 | 1.05E-01 | 6.27E+00 |
| GO:0016051~carbohydrate biosynthetic process | 6 | 1.30E+01 | 7.74E-03 | 4.64E+00 | 7.73E-01 | 1.38E-01 | 9.21E+00 |
| GO:0015847~putrescine transport | 2 | 4.35E+00 | 8.12E-03 | 2.39E+02 | 7.89E-01 | 1.32E-01 | 9.64E+00 |
| GO:0006571~tyrosine biosynthetic process | 2 | 4.35E+00 | 1.01E-02 | 1.91E+02 | 8.57E-01 | 1.50E-01 | 1.19E+01 |
| GO:0006570~tyrosine metabolic process | 2 | 4.35E+00 | 1.01E-02 | 1.91E+02 | 8.57E-01 | 1.50E-01 | 1.19E+01 |
| GO:0009073~aromatic amino acid family biosynthetic process | 3 | 6.52E+00 | 1.04E-02 | 1.88E+01 | 8.65E-01 | 1.43E-01 | 1.22E+01 |
| GO:0046417~chorismate metabolic process | 3 | 6.52E+00 | 1.07E-02 | 1.86E+01 | 8.72E-01 | 1.36E-01 | 1.25E+01 |
| GO:0009072~aromatic amino acid family metabolic process | 3 | 6.52E+00 | 1.18E-02 | 1.77E+01 | 8.96E-01 | 1.40E-01 | 1.37E+01 |
| GO:0046394~carboxylic acid biosynthetic process | 6 | 1.30E+01 | 1.18E-02 | 4.18E+00 | 8.97E-01 | 1.33E-01 | 1.38E+01 |
| GO:0016053~organic acid biosynthetic process | 6 | 1.30E+01 | 1.21E-02 | 4.16E+00 | 9.01E-01 | 1.27E-01 | 1.40E+01 |
| GO:0009094~L-phenylalanine biosynthetic process | 2 | 4.35E+00 | 1.42E-02 | 1.36E+02 | 9.34E-01 | 1.40E-01 | 1.63E+01 |
| GO:0006558~L-phenylalanine metabolic process | 2 | 4.35E+00 | 1.42E-02 | 1.36E+02 | 9.34E-01 | 1.40E-01 | 1.63E+01 |
| GO:0009095~aromatic amino acid family biosynthetic process, prephenate pathway | 2 | 4.35E+00 | 1.62E-02 | 1.19E+02 | 9.56E-01 | 1.51E-01 | 1.84E+01 |
| GO:0009628~response to abiotic stimulus | 3 | 6.52E+00 | 1.69E-02 | 1.46E+01 | 9.62E-01 | 1.50E-01 | 1.91E+01 |
| GO:0009084~glutamine family amino acid biosynthetic process | 3 | 6.52E+00 | 2.28E-02 | 1.25E+01 | 9.88E-01 | 1.89E-01 | 2.50E+01 |
| GO:0000271~polysaccharide biosynthetic process | 5 | 1.09E+01 | 2.32E-02 | 4.42E+00 | 9.89E-01 | 1.85E-01 | 2.54E+01 |
| GO:0005978~glycogen biosynthetic process | 2 | 4.35E+00 | 2.42E-02 | 7.96E+01 | 9.91E-01 | 1.84E-01 | 2.62E+01 |
| GO:0006006~glucose metabolic process | 4 | 8.70E+00 | 3.40E-02 | 5.44E+00 | 9.99E-01 | 2.41E-01 | 3.50E+01 |
| GO:0005996~monosaccharide metabolic process | 5 | 1.09E+01 | 3.83E-02 | 3.78E+00 | 9.99E-01 | 2.58E-01 | 3.85E+01 |
| GO:0015846~polyamine transport | 2 | 4.35E+00 | 3.99E-02 | 4.77E+01 | 1.00E+00 | 2.59E-01 | 3.98E+01 |
| GO:0006796~phosphate metabolic process | 4 | 8.70E+00 | 5.25E-02 | 4.57E+00 | 1.00E+00 | 3.17E-01 | 4.89E+01 |
| GO:0005976~polysaccharide metabolic process | 5 | 1.09E+01 | 5.52E-02 | 3.35E+00 | 1.00E+00 | 3.21E-01 | 5.06E+01 |
| GO:0044264~cellular polysaccharide metabolic process | 4 | 8.70E+00 | 5.63E-02 | 4.44E+00 | 1.00E+00 | 3.17E-01 | 5.13E+01 |
| GO:0019318~hexose metabolic process | 4 | 8.70E+00 | 6.18E-02 | 4.27E+00 | 1.00E+00 | 3.34E-01 | 5.48E+01 |
| GO:0006793~phosphorus metabolic process | 4 | 8.70E+00 | 6.31E-02 | 4.23E+00 | 1.00E+00 | 3.31E-01 | 5.56E+01 |
| GO:0019438~aromatic compound biosynthetic process | 3 | 6.52E+00 | 6.50E-02 | 6.99E+00 | 1.00E+00 | 3.31E-01 | 5.67E+01 |
| GO:0006596~polyamine biosynthetic process | 2 | 4.35E+00 | 6.89E-02 | 2.73E+01 | 1.00E+00 | 3.38E-01 | 5.88E+01 |
| GO:0034637~cellular carbohydrate biosynthetic process | 4 | 8.70E+00 | 7.35E-02 | 3.97E+00 | 1.00E+00 | 3.49E-01 | 6.13E+01 |
| GO:0009089~lysine biosynthetic process via diaminopimelate | 2 | 4.35E+00 | 8.21E-02 | 2.27E+01 | 1.00E+00 | 3.73E-01 | 6.55E+01 |
| GO:0046451~diaminopimelate metabolic process | 2 | 4.35E+00 | 8.21E-02 | 2.27E+01 | 1.00E+00 | 3.73E-01 | 6.55E+01 |
| GO:0006553~lysine metabolic process | 2 | 4.35E+00 | 8.21E-02 | 2.27E+01 | 1.00E+00 | 3.73E-01 | 6.55E+01 |
| GO:0009085~lysine biosynthetic process | 2 | 4.35E+00 | 8.21E-02 | 2.27E+01 | 1.00E+00 | 3.73E-01 | 6.55E+01 |
| GO:0009250~glucan biosynthetic process | 2 | 4.35E+00 | 8.58E-02 | 2.17E+01 | 1.00E+00 | 3.79E-01 | 6.73E+01 |
| GO:0006595~polyamine metabolic process | 2 | 4.35E+00 | 9.88E-02 | 1.87E+01 | 1.00E+00 | 4.16E-01 | 7.26E+01 |
| GO:0009064~glutamine family amino acid metabolic process | 3 | 6.52E+00 | 9.91E-02 | 5.47E+00 | 1.00E+00 | 4.08E-01 | 7.27E+01 |

**Supplementary Table S11. The mutations accumulated in the six evolved strains of *E. coli*** during adaptive evolution for 2496 hours in the presence of ethanol.

| Functional role in fitness or ethanol tolerance | Strain | Gene | Mutation in genome | Effect on protein sequence | Gene expression |
| --- | --- | --- | --- | --- | --- |
|  |  |  |  |  |  |
| energy saving | Strain A | radA, nadR, slp, insH, yhiS | deletion | knockout | no expression in all six strains |
|  | strain B | yeaR | in-frame IS5 insertion; 1-hydroxy-2-methyl-2-(E)-butenyl 4-diphosphate synthase | knockout | no expression in all six strains |
|  | strain B | ilvG (pseudogene) | A974T | Leu321stop | consistently downregulation in all six strains |
|  | Strain C | deletion of 12 genes | insH nmpC essD ybcS rzpD rzoD borD ybcV ybcW nohB tfaD ybcY | | 12 genes are not expressed in all six strains |
|  | Strain F | wzxC | A1304T |  | no expression |
|  | Strain F | miaB | 728 deletion -88 | knockout | no expression |
|  | Strain E | zapE (yhcM) | 739: +C | insertion of C causes frameshift from 247 amino acid | consistent upregulation in all six strains |
|  | Strain D | nanC (yjhA) | IS5 insertion at promoter (-39) | not expressed | not exprssed |
|  | Strain C | nagE | 864 +3:CCG | 289Pro (helical disruption beween 282-302) | consistent downregulation in all six strains |
|  | Strain C | menC | 485 IS186 (1343bp) insertion | knockout | consistent downregulation in all six strains |
|  | Strain D | ispG | T992C | Glu331Gly | expressed low and no apparent fold change in six strains |
| energy generation | Strain E | atpE | T54G | Gly18Gly in helical region (11-31) | consistent downregulation in all six strains, which determine the proton stiochiometry for ATP production |
| Reduced stress response | Strain D | proQ | A272T | Leu91Gln in critical region | consistent regulation in all six strains |
|  | Strain E | cspC | 45 IS5 insertion | knockout | expressed high with downregulation in some strains |
|  | Strain F | cspC | 45 IS5 insertion | knockout | expressed high with downregulation in some strains |
|  | strain A | hns | promoter insertion | promoter | consistently upregulation in all six strains |
|  | strain B | hns | promoter insertion | promoter | consistently upregulation in all six strains |
|  | Strain C | hns | promoter insertion | promoter | consistently upregulation in all six strains |
|  | Strain D | hns | promoter insertion | promoter | consistently upregulation in all six strains |
|  | Strain E | hns | promoter insertion | promoter | consistently upregulation in all six strains |
| Increased SOS/cold shock | strain A | hns | promoter insertion | promoter | consistently upregulation in all six strains |
|  | strain B | hns | promoter insertion | promoter | consistently upregulation in all six strains |
|  | Strain C | hns | promoter insertion | promoter | consistently upregulation in all six strains |
|  | Strain D | hns | promoter insertion | promoter | consistently upregulation in all six strains |
|  | Strain E | hns | promoter insertion | promoter | consistently upregulation in all six strains |
|  | Strain C | relA | A1547G | Lys516Pro in not a critical region | downregulation in strain C at first three time point but not latter two; |
|  | Strain E | relA | A1364T | Leu455Gln in a TGS domain | downregulation in strain C at first three time point but not latter two; |
|  | Strain F | relA | T417G | Leu139Phe in HD domain | downregulation in strain C at first three time point but not latter two; |
| Gene regulation | Strain C | rpoC | G2819A | Ala940Val in the cleft region (domain 5) of RNA polymerase β' subunit | minor upregulation compared to other strains |
|  | Strain C | rpoA | T961A | Trp321Gly in a not required domain | minor upregulation compared to other strains but expressed at high levels |
|  | Strain D | rpsD | T226G | Tyr76Asp | expressed high with no apparent fold change |
|  | strain B | iscR | T320A | His107Leu in HTH rrf2-type domain | expression increases after mutation |
|  | Strain F | iscR | T320A | His107Leu in HTH rrf2-type domain | expression increases after mutation |
|  | Strain E | zapE (yhcM) | 739: +C | insertion of C causes frameshift from 247 amino acid | consistent upregulation in all six strains |
| Unkown | Strain C | yeaY | T168A | A56A | consistent upregulation in all six strains |
| Increased in biosynthesis | Strain C | duplication | 220 Kb (180 genes) duplication | upregulation in 96 genes |  |

**Supplementary Table S12. The genes we identified in the duplicated fragment (220 kb) of strain C.**

(The long list is put in a separate spreadsheet.)

**Supplementary Table S13. The functional enrichment of the genes in the duplicated fragment of strain C.**

| Term | Count | % | PValue | Fold Enrichment | Bonferroni | Benjamini | FDR |
| --- | --- | --- | --- | --- | --- | --- | --- |
| GO:0016052~carbohydrate catabolic process | 26 | 14.69 | 7.79E-13 | 5.97 | 2.60E-10 | 2.60E-10 | 1.06E-09 |
| GO:0046401~lipopolysaccharide core region metabolic process | 9 | 5.08 | 4.55E-09 | 22.95 | 1.52E-06 | 7.60E-07 | 6.18E-06 |
| GO:0009244~lipopolysaccharide core region biosynthetic process | 9 | 5.08 | 4.55E-09 | 22.95 | 1.52E-06 | 7.60E-07 | 6.18E-06 |
| GO:0009312~oligosaccharide biosynthetic process | 9 | 5.08 | 1.96E-08 | 19.18 | 6.55E-06 | 2.18E-06 | 2.66E-05 |
| GO:0009311~oligosaccharide metabolic process | 9 | 5.08 | 1.30E-07 | 15.12 | 4.36E-05 | 1.09E-05 | 1.77E-04 |
| GO:0033692~cellular polysaccharide biosynthetic process | 14 | 7.91 | 4.85E-06 | 4.92 | 0.001619 | 3.24E-04 | 0.006588 |
| GO:0034637~cellular carbohydrate biosynthetic process | 15 | 8.47 | 6.14E-06 | 4.45 | 0.002049 | 3.42E-04 | 0.008339 |
| GO:0044264~cellular polysaccharide metabolic process | 14 | 7.91 | 9.00E-06 | 4.65 | 0.003003 | 4.30E-04 | 0.012228 |
| GO:0009103~lipopolysaccharide biosynthetic process | 12 | 6.78 | 1.22E-05 | 5.44 | 0.004051 | 5.07E-04 | 0.016506 |
| GO:0008653~lipopolysaccharide metabolic process | 12 | 6.78 | 1.58E-05 | 5.29 | 0.005265 | 5.86E-04 | 0.021464 |
| GO:0016051~carbohydrate biosynthetic process | 15 | 8.47 | 9.51E-05 | 3.47 | 0.031281 | 0.003173 | 0.129147 |
| GO:0000271~polysaccharide biosynthetic process | 14 | 7.91 | 9.54E-05 | 3.70 | 0.031379 | 0.002894 | 0.129558 |
| GO:0005976~polysaccharide metabolic process | 16 | 9.04 | 1.21E-04 | 3.21 | 0.039473 | 0.00335 | 0.163631 |
| GO:0008610~lipid biosynthetic process | 13 | 7.34 | 1.78E-04 | 3.73 | 0.057779 | 0.004568 | 0.241717 |
| GO:0006261~DNA-dependent DNA replication | 7 | 3.95 | 4.85E-04 | 6.99 | 0.149592 | 0.011508 | 0.656743 |

**Supplementary Table S14. The differential expression of ethanol tolerance genes in the evolved strains.**

(The table is too big to fit in here so is put in a separate spreadsheet.)

**Supplementary Table S**15. The functional enrichment of the differentially expressed ethanol tolerance genes.

| Term | Count | % | PValue | Fold Enrichment | Bonferroni | Benjamini | FDR |
| --- | --- | --- | --- | --- | --- | --- | --- |
| GO:0006412~translation | 46 | 12.01 | 6.50E-31 | 9.58 | 3.90E-28 | 3.90E-28 | 9.60E-28 |
| GO:0044271~nitrogen compound biosynthetic process | 70 | 18.28 | 5.44E-18 | 3.17 | 3.26E-15 | 1.63E-15 | 8.04E-15 |
| GO:0046394~carboxylic acid biosynthetic process | 50 | 13.05 | 6.54E-16 | 3.85 | 4.00E-13 | 1.33E-13 | 9.88E-13 |
| GO:0016053~organic acid biosynthetic process | 50 | 13.05 | 8.67E-16 | 3.83 | 5.33E-13 | 1.33E-13 | 1.31E-12 |
| GO:0009309~amine biosynthetic process | 43 | 11.23 | 2.12E-13 | 3.78 | 1.27E-10 | 2.54E-11 | 3.13E-10 |
| GO:0008652~cellular amino acid biosynthetic process | 39 | 10.18 | 4.87E-12 | 3.74 | 2.92E-09 | 4.87E-10 | 7.20E-09 |
| GO:0034660~ncRNA metabolic process | 28 | 7.31 | 3.32E-09 | 3.91 | 1.99E-06 | 2.84E-07 | 4.90E-06 |
| GO:0018130~heterocycle biosynthetic process | 29 | 7.57 | 4.07E-09 | 3.76 | 2.44E-06 | 3.05E-07 | 6.01E-06 |
| GO:0006024~glycosaminoglycan biosynthetic process | 14 | 3.66 | 4.99E-09 | 8.89 | 3.00E-06 | 3.33E-07 | 7.37E-06 |
| GO:0006023~aminoglycan biosynthetic process | 14 | 3.66 | 4.99E-09 | 8.89 | 3.00E-06 | 3.33E-07 | 7.37E-06 |
| GO:0009252~peptidoglycan biosynthetic process | 14 | 3.66 | 4.99E-09 | 8.89 | 3.00E-06 | 3.33E-07 | 7.37E-06 |
| GO:0044038~cell wall macromolecule biosynthetic process | 14 | 3.66 | 7.81E-09 | 8.58 | 4.69E-06 | 4.69E-07 | 1.15E-05 |
| GO:0070589~cellular component macromolecule biosynthetic process | 14 | 3.66 | 7.81E-09 | 8.58 | 4.69E-06 | 4.69E-07 | 1.15E-05 |
| GO:0010382~cellular cell wall macromolecule metabolic process | 14 | 3.66 | 9.03E-09 | 8.48 | 5.42E-06 | 4.92E-07 | 1.33E-05 |
| GO:0009273~peptidoglycan-based cell wall biogenesis | 14 | 3.66 | 2.36E-08 | 7.85 | 1.42E-05 | 1.18E-06 | 3.49E-05 |
| GO:0042546~cell wall biogenesis | 14 | 3.66 | 2.36E-08 | 7.85 | 1.42E-05 | 1.18E-06 | 3.49E-05 |
| GO:0009451~RNA modification | 18 | 4.70 | 1.48E-07 | 4.94 | 8.86E-05 | 6.81E-06 | 2.18E-04 |
| GO:0006399~tRNA metabolic process | 21 | 5.48 | 1.86E-07 | 4.15 | 1.12E-04 | 7.98E-06 | 2.75E-04 |
| GO:0051186~cofactor metabolic process | 35 | 9.14 | 2.79E-07 | 2.70 | 1.67E-04 | 1.11E-05 | 4.11E-04 |
| GO:0019438~aromatic compound biosynthetic process | 17 | 4.44 | 1.86E-06 | 4.37 | 0.001118 | 6.99E-05 | 0.002753 |
| GO:0030203~glycosaminoglycan metabolic process | 17 | 4.44 | 3.91E-06 | 4.13 | 0.002345 | 1.38E-04 | 0.005779 |
| GO:0000270~peptidoglycan metabolic process | 17 | 4.44 | 3.91E-06 | 4.13 | 0.002345 | 1.38E-04 | 0.005779 |
| GO:0042364~water-soluble vitamin biosynthetic process | 18 | 4.70 | 4.20E-06 | 3.89 | 0.002515 | 1.40E-04 | 0.006199 |
| GO:0006022~aminoglycan metabolic process | 17 | 4.44 | 5.24E-06 | 4.04 | 0.003137 | 1.65E-04 | 0.007733 |
| GO:0006790~sulfur metabolic process | 18 | 4.70 | 1.25E-05 | 3.58 | 0.007478 | 3.75E-04 | 0.018475 |
| GO:0034470~ncRNA processing | 19 | 4.96 | 1.72E-05 | 3.34 | 0.010294 | 4.93E-04 | 0.025468 |
| GO:0051188~cofactor biosynthetic process | 24 | 6.27 | 1.78E-05 | 2.80 | 0.010614 | 4.85E-04 | 0.026263 |
| GO:0043039~tRNA aminoacylation | 10 | 2.61 | 1.78E-05 | 6.67 | 0.010617 | 4.64E-04 | 0.02627 |
| GO:0043038~amino acid activation | 10 | 2.61 | 1.78E-05 | 6.67 | 0.010617 | 4.64E-04 | 0.02627 |
| GO:0006418~tRNA aminoacylation for protein translation | 10 | 2.61 | 1.78E-05 | 6.67 | 0.010617 | 4.64E-04 | 0.02627 |
| GO:0016051~carbohydrate biosynthetic process | 29 | 7.57 | 1.81E-05 | 2.48 | 0.010775 | 4.51E-04 | 0.026663 |
| GO:0015949~nucleobase, nucleoside and nucleotide interconversion | 9 | 2.35 | 1.85E-05 | 7.78 | 0.011011 | 4.43E-04 | 0.027251 |
| GO:0006767~water-soluble vitamin metabolic process | 18 | 4.70 | 2.11E-05 | 3.44 | 0.012592 | 4.87E-04 | 0.031188 |
| GO:0009110~vitamin biosynthetic process | 18 | 4.70 | 2.53E-05 | 3.39 | 0.015084 | 5.63E-04 | 0.037407 |
| GO:0000271~polysaccharide biosynthetic process | 26 | 6.79 | 3.73E-05 | 2.54 | 0.022104 | 7.98E-04 | 0.055006 |
| GO:0008360~regulation of cell shape | 10 | 2.61 | 3.88E-05 | 6.06 | 0.023039 | 8.03E-04 | 0.057358 |
| GO:0022604~regulation of cell morphogenesis | 10 | 2.61 | 3.88E-05 | 6.06 | 0.023039 | 8.03E-04 | 0.057358 |
| GO:0009084~glutamine family amino acid biosynthetic process | 11 | 2.87 | 6.55E-05 | 5.04 | 0.038555 | 0.00131 | 0.096736 |
| GO:0006396~RNA processing | 20 | 5.22 | 6.59E-05 | 2.90 | 0.038764 | 0.001275 | 0.09727 |
| GO:0044272~sulfur compound biosynthetic process | 14 | 3.66 | 6.87E-05 | 3.88 | 0.040359 | 0.001287 | 0.101355 |
| GO:0006766~vitamin metabolic process | 18 | 4.70 | 9.78E-05 | 3.04 | 0.056991 | 0.001777 | 0.144337 |
| GO:0006400~tRNA modification | 8 | 2.09 | 1.19E-04 | 7.15 | 0.068849 | 0.002096 | 0.175436 |
| GO:0008610~lipid biosynthetic process | 23 | 6.01 | 2.11E-04 | 2.43 | 0.118935 | 0.003611 | 0.311206 |
| GO:0005976~polysaccharide metabolic process | 29 | 7.57 | 2.13E-04 | 2.15 | 0.120035 | 0.003546 | 0.31427 |
| GO:0007049~cell cycle | 11 | 2.87 | 3.95E-04 | 4.05 | 0.210902 | 0.006381 | 0.581356 |
| GO:0022613~ribonucleoprotein complex biogenesis | 10 | 2.61 | 4.89E-04 | 4.36 | 0.254277 | 0.007691 | 0.719617 |
| GO:0042254~ribosome biogenesis | 10 | 2.61 | 4.89E-04 | 4.36 | 0.254277 | 0.007691 | 0.719617 |
| GO:0009628~response to abiotic stimulus | 9 | 2.35 | 5.39E-04 | 4.84 | 0.276452 | 0.008263 | 0.793364 |
| GO:0034404~nucleobase, nucleoside and nucleotide biosynthetic process | 17 | 4.44 | 5.74E-04 | 2.72 | 0.291385 | 0.008574 | 0.844276 |
| GO:0034654~nucleobase, nucleoside, nucleotide and nucleic acid biosynthetic process | 17 | 4.44 | 5.74E-04 | 2.72 | 0.291385 | 0.008574 | 0.844276 |
| GO:0006732~coenzyme metabolic process | 21 | 5.48 | 6.22E-04 | 2.37 | 0.311664 | 0.009068 | 0.915119 |
| GO:0043241~protein complex disassembly | 5 | 1.31 | 7.01E-04 | 11.98 | 0.343594 | 0.009973 | 1.0309 |
| GO:0032984~macromolecular complex disassembly | 5 | 1.31 | 7.01E-04 | 11.98 | 0.343594 | 0.009973 | 1.0309 |
| GO:0022411~cellular component disassembly | 5 | 1.31 | 7.01E-04 | 11.98 | 0.343594 | 0.009973 | 1.0309 |
| GO:0034623~cellular macromolecular complex disassembly | 5 | 1.31 | 7.01E-04 | 11.98 | 0.343594 | 0.009973 | 1.0309 |
| GO:0043624~cellular protein complex disassembly | 5 | 1.31 | 7.01E-04 | 11.98 | 0.343594 | 0.009973 | 1.0309 |

**Supplementary Table S**16. The correlation between DNA replication and biosynthetic processes in EA samples.

| Biosynthetic pathway | DNA replication |
| --- | --- |
| arginine biosynthetic process | 0.531908752 |
| O antigen biosynthetic process | 0.517878845 |
| tetrahydrofolate biosynthetic process | 0.488355868 |
| folic acid-containing compound biosynthetic process | 0.468007707 |
| peptidoglycan biosynthetic process | 0.462150141 |
| branched-chain amino acid biosynthetic process | 0.461755766 |
| polysaccharide biosynthetic process | 0.447272495 |
| cell wall macromolecule biosynthetic process | 0.44262795 |
| aromatic amino acid family biosynthetic process | 0.439597622 |
| fatty acid biosynthetic process | 0.436429604 |
| carbohydrate biosynthetic process | 0.4353602 |
| IMP biosynthetic process | 0.434460821 |
| lipopolysaccharide biosynthetic process | 0.428366702 |
| methionine biosynthetic process | 0.421415932 |
| lipopolysaccharide core region biosynthetic process | 0.411290378 |
| valine biosynthetic process | 0.411037737 |
| ubiquinone biosynthetic process | 0.405944823 |
| lipid A biosynthetic process | 0.397855994 |
| purine ribonucleoside monophosphate biosynthetic process | 0.396236634 |
| lysine biosynthetic process | 0.383601607 |
| lysine biosynthetic process via diaminopimelate | 0.370373966 |
| glycerophospholipid biosynthetic process | 0.337257045 |
| ribonucleoside monophosphate biosynthetic process | 0.329281 |
| phospholipid biosynthetic process | 0.318954784 |
| lipid biosynthetic process | 0.318812723 |
| cofactor biosynthetic process | 0.316265294 |
| phosphatidic acid biosynthetic process | 0.310214875 |

**Supplementary Table S**17. The correlation between PTS system and some biosynthetic processes in EA samples.

| **Biosynthetic pathways** | **PTS** |
| --- | --- |
| arginine biosynthetic process | -0.32 |
| purine ribonucleoside monophosphate biosynthetic process | -0.30 |
| IMP biosynthetic process | -0.30 |
| ribonucleoside monophosphate biosynthetic process | -0.30 |
| porphyrin-containing compound biosynthetic process | -0.29 |
| aromatic amino acid family biosynthetic process | -0.27 |
| lipopolysaccharide core region biosynthetic process | -0.27 |
| ubiquinone biosynthetic process | -0.25 |

**Supplementary Table S**18. Functions of differentially expressed genes in SOS response in EA samples.

| Level | Gene | Gene function |
| --- | --- | --- |
| Transcription | lexA | LexA DNA-binding transcriptional repressor |
| DNA repair | recN | protein used in recombination and DNA repair for breaks occuromg at two or more locations, |
|  | uvrD | ssDNA translocase and dsDNA helicase - DNA helicase II |
|  | cho | endonuclease of nucleotide excision repair |
|  | dinG | ATP-dependent helicase |
|  | dinB | DNA polymerase IV (Y-family DNA polymerase; translesion DNA synthesis) |
|  | ssb | ssDNA-binding protein, protects ssDNA from degradation |
|  | sulA | SOS cell division inhibitor |
| Other | ybfE | LexA-regulated protein, response to DNA damaging agent mitomycin C |
|  | yebG | conserved protein regulated by LexA, (DNA damage-inducible) |
|  | dinF | putative transport protein, multi antimicrobial extrusion (MATE) family (DNA damage (UV or mitomycin C) inducible) |

**Supplementary Table S**19. Functions of differentially expressed genes in cold shock response in EA samples.

| Level | Gene | Gene function |
| --- | --- | --- |
| DNA integrity | recA | DNA strand exchange and recombination protein with protease and nuclease activity |
|  | gyrA | DNA gyrase, subunit A |
| Transcription | nusA | transcription termination/antitermination L factor |
|  | cspA | CspA DNA-binding transcriptional activator |
|  | hns | H-NS DNA-binding transcriptional dual regulator |
|  | pnp | polynucleotide phosphorylase, 3' to 5' exonuclease and a 3'-terminal oligonucleotide polymerase |
|  | cspE | transcription antiterminator and regulator of RNA stability |
| Translation | infB | protein chain initiation factor IF2 |
|  | tig | chaperone protein Tig |
|  | rbfA | 30S ribosome binding factor |
|  | deaD | DEAD-box RNA helicase, assembly of the large subunit of the ribosome |
|  | hscB | co-chaperone for [Fe-S] cluster biosynthesis |
|  | hscA | co-chaperone for [Fe-S] cluster biosynthesis |
| Other | cspG | cold shock protein CspG |

# Supplementary Figures

**Supplementary Fig. S1. Differentially expressed genes in response pathways to acid stress, osmotic stress and trehalose production, envelope stress, and heat shock response in NEA samples of *E. coli* strains in the presence of ethanol. *x*-Axis indicates the samples used in our analysis and *y-*axis indicates gene names (same hereafter for other figures).**


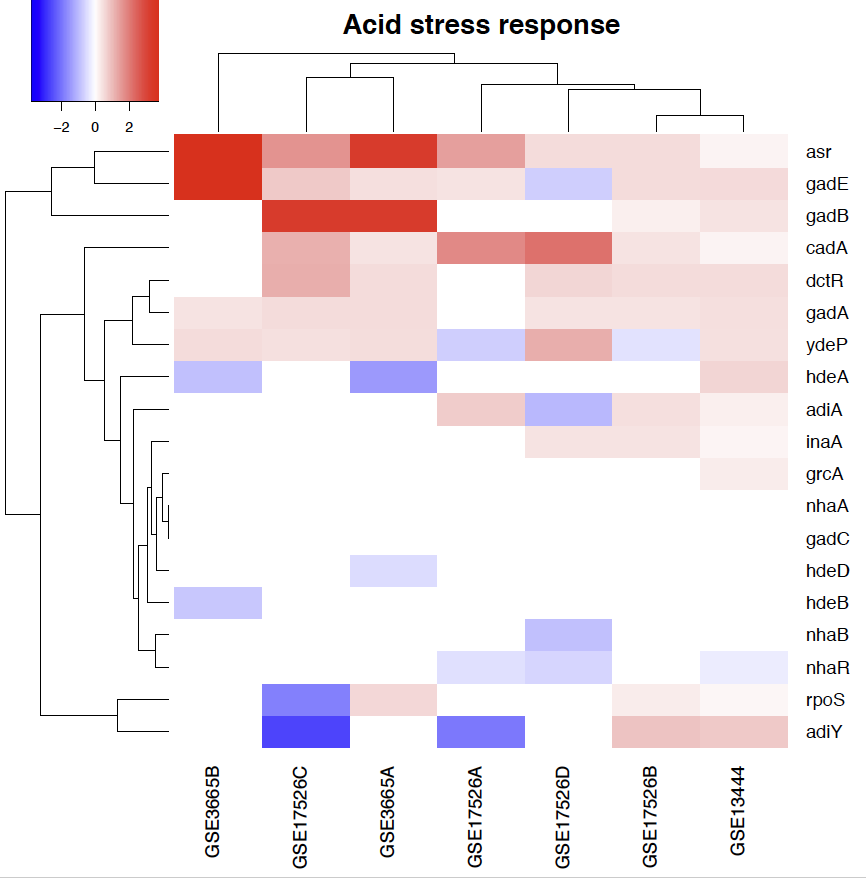


**Supplementary Fig. S2. Differential expression of genes in ArcAB regulon in response to decreased intracellular oxygen level in NEA samples of *E. coli* strains.**


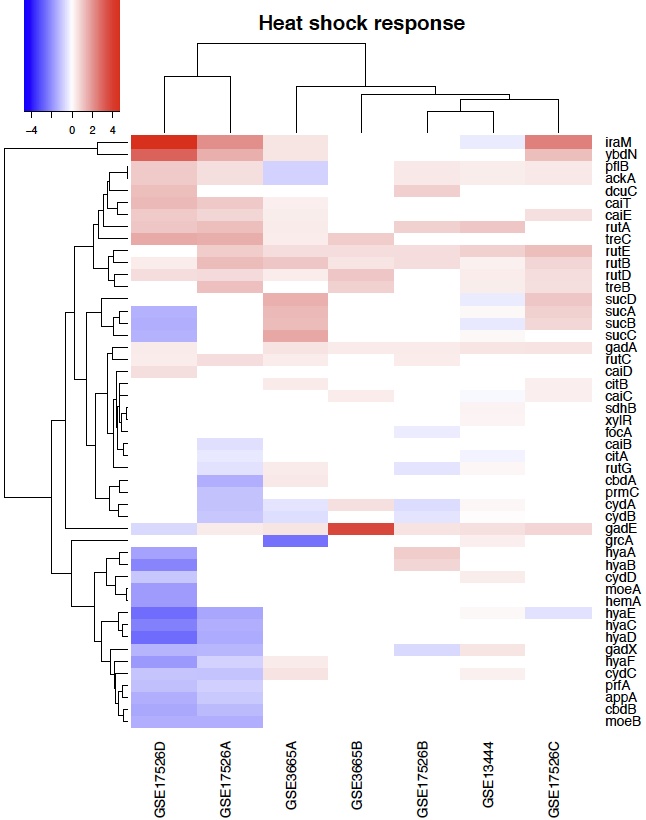


**Supplementary Fig. S3. The energy taxis signaling components in *E. coli*. The green-coded proteins are available in *E. coli*. The Figure is drawn from KEGG.**


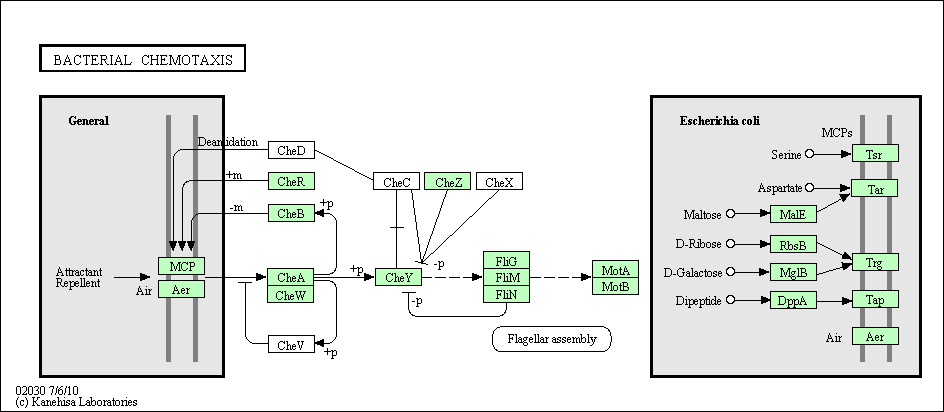


**Supplementary Fig. S4. Differential expression of genes involved in flagellum assembly following signal transduction in NEA samples of *E. coli* strains.**


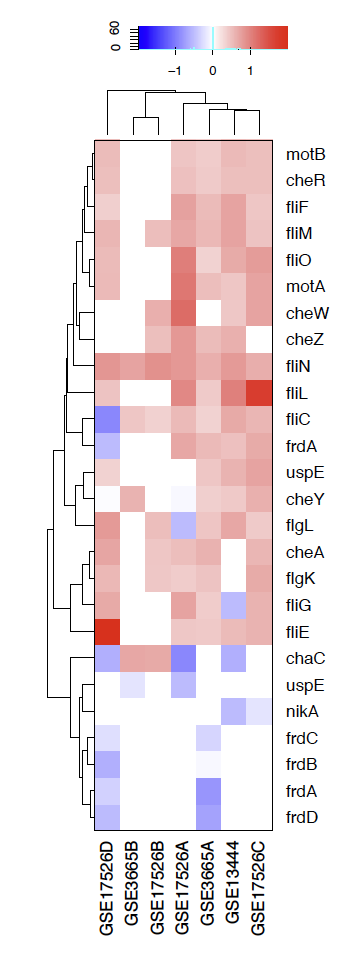


**Supplementary Fig. S5. Down-regulation of genes involved in the three stages in peptidoglycan biosynthesis in the NEA samples. (A) initiation. (B) crosslinking with peptides. (C) maturation. The x-axis is for samples and the y-axis represents genes in each panel.**


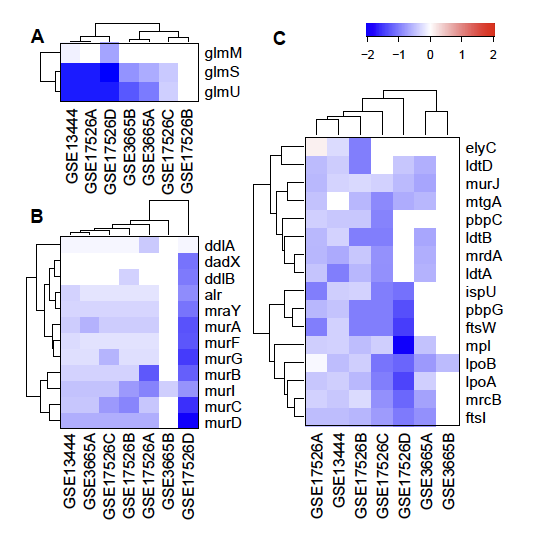


**Supplementary Fig. S6. Stage one of peptidoglycan biosynthesis, initiation, in *E. coli*.**


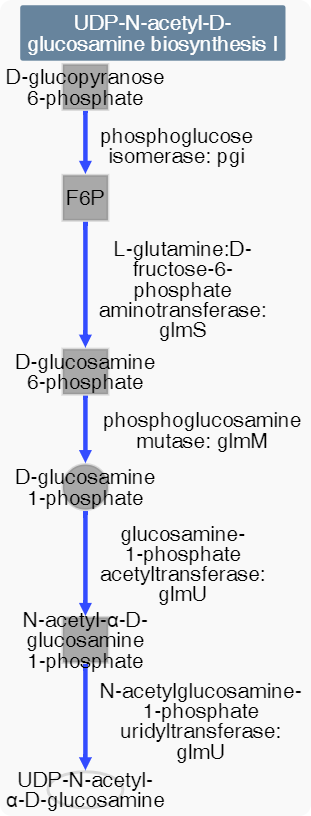


**Supplementary Fig. S7. Stage two of peptidoglycan biosynthesis, crosslinking, in *E. coli*.**


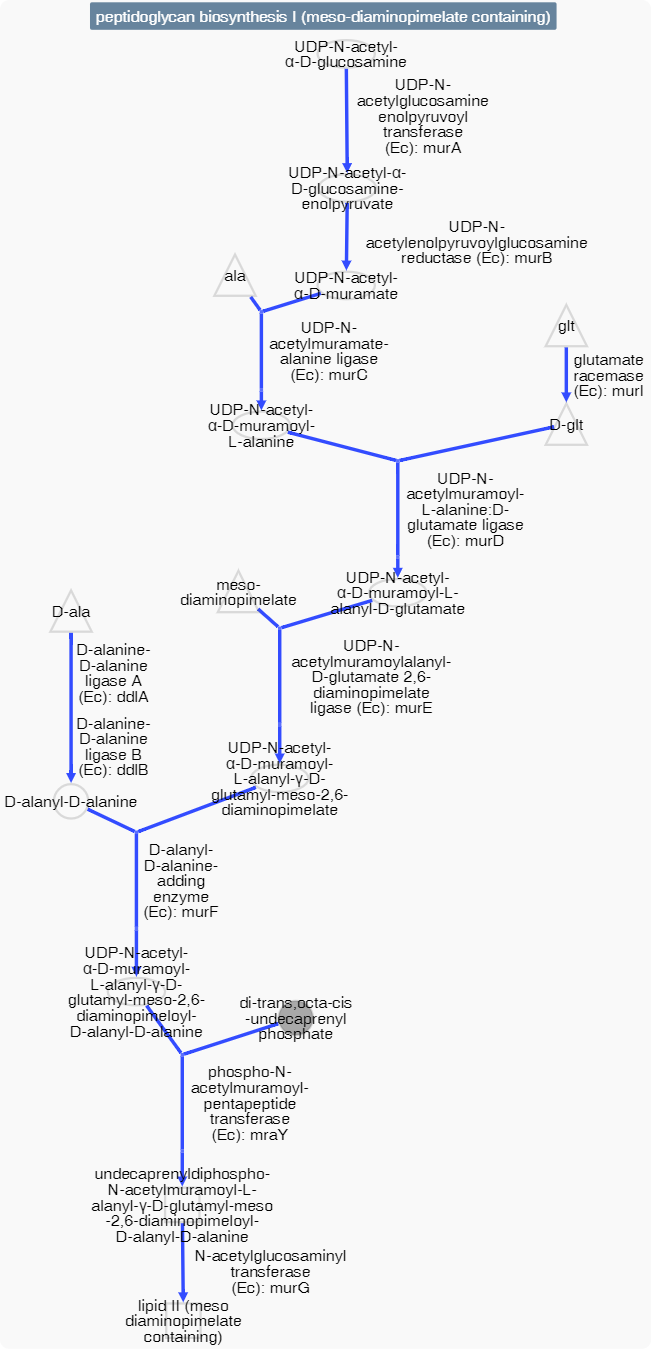


**Supplementary Fig. S8. Stage three of peptidoglycan biosynthesis, maturation, in *E. coli*.**


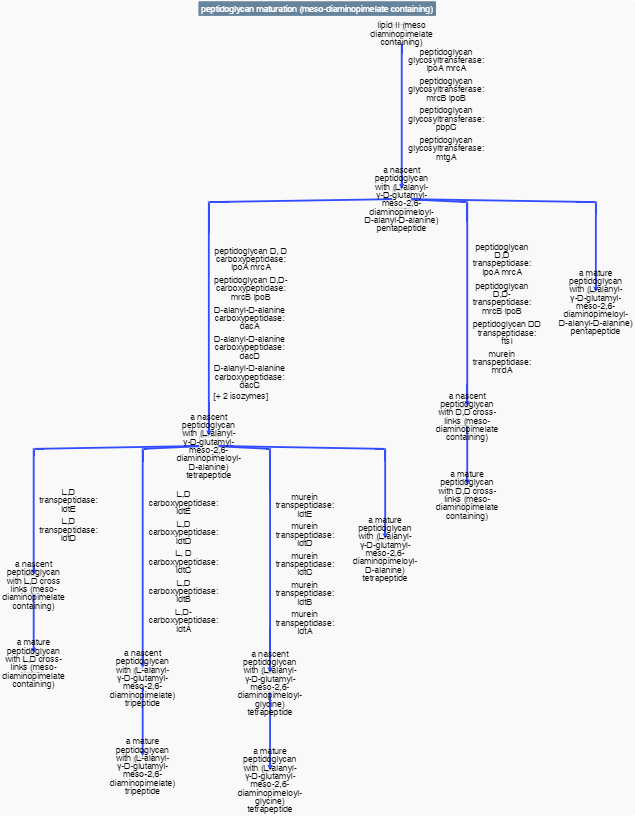


**Supplementary Fig. S9. The quantity and functional classification of mutated genes in six EA strains. A: the number and type of mutations in each evolved strain; B: the number of mutated genes involved in each functional category.**


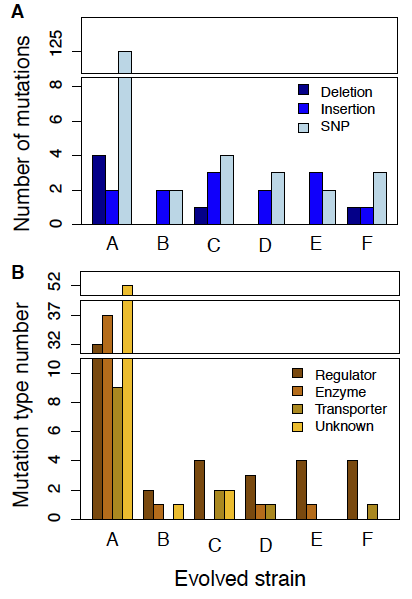


**Supplementary Fig. S10. The expression profiles of 180 duplicated genes in the evolved strain C. A: expression levels of each gene; B: the expression level of duplicated genes compared between time points and asterisk indicates significance in Wilcoxon test at *p* < 0.001; and C: the differential expression of the duplicated genes in all six evolved strains at five time points.**


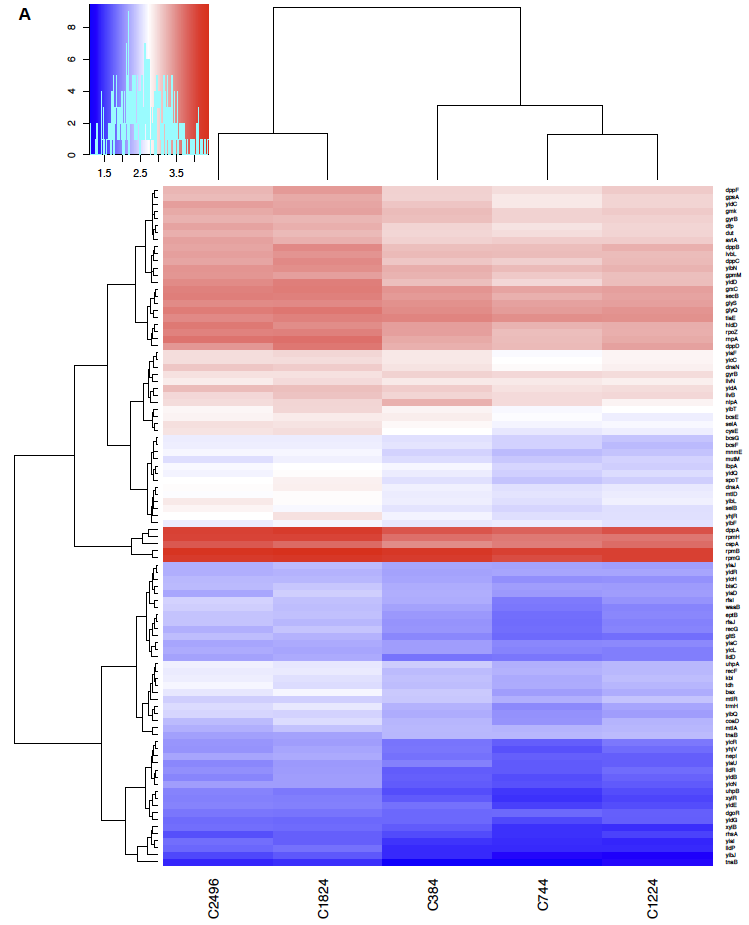


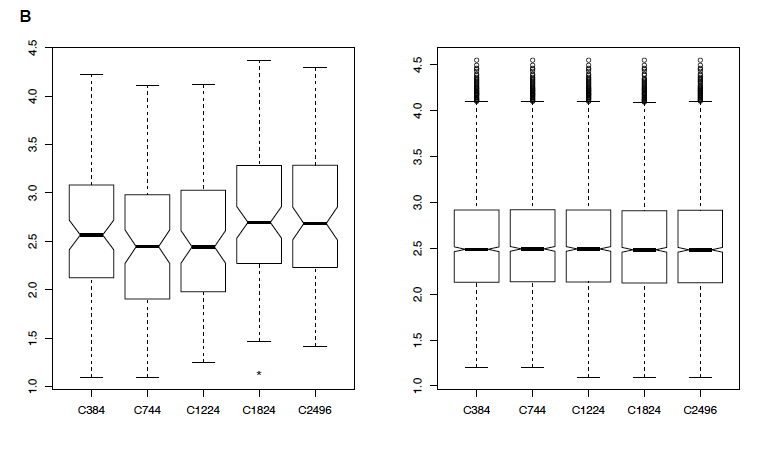


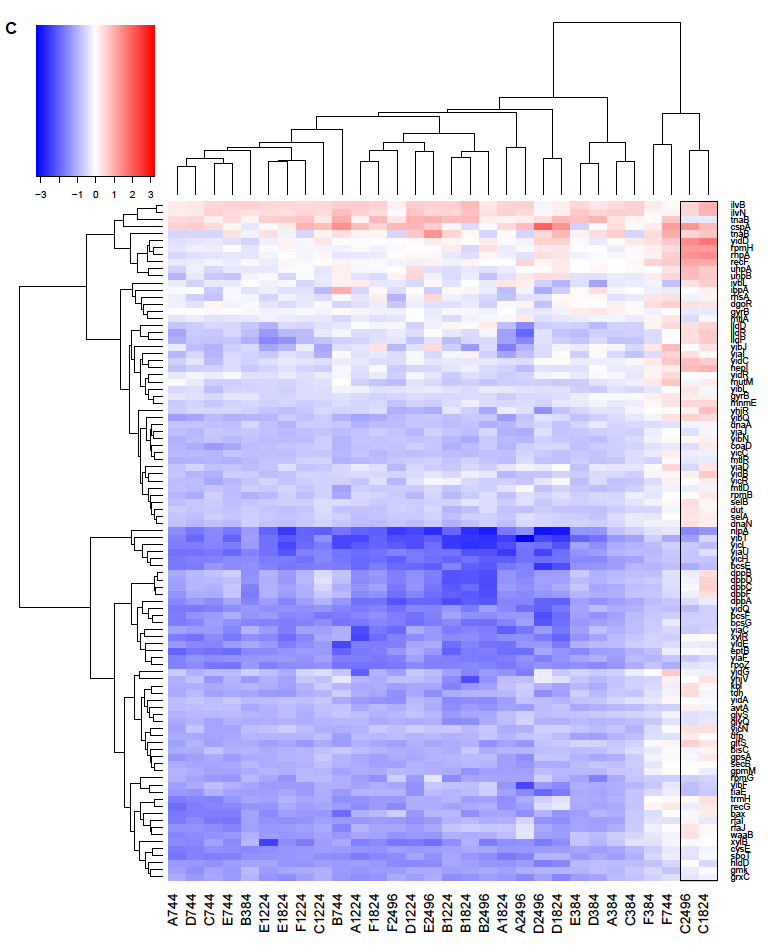


**Figures S11. The expression profiles of ethanol tolerance genes identified through experimental overexpression or knockout. The heat map is followed by first consistently upregulated genes and then consistently downregulated genes over the course of evolution.**


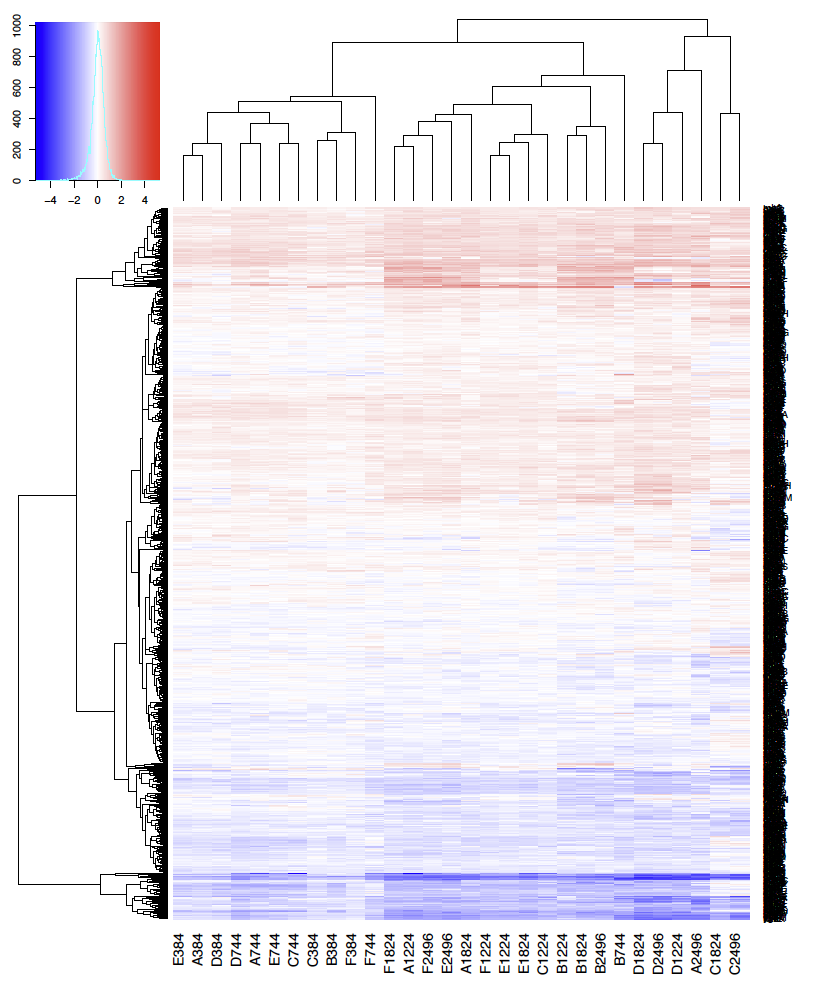


**Supplementary Fig. S12. The differential gene expression of SoxRS and OxyR regulons in the evolved strains, which are each followed by the consistently up- or down-regulated genes. (A) and (C): average level of gene expression in SoxRS and OxyrR regulon; (B) and (D): differential gene expression in SoxRS and OxyrR regulon; (C) and (F): consistently up- or down-regulated genes in SoxRS and OxyrR regulon during evolution.**


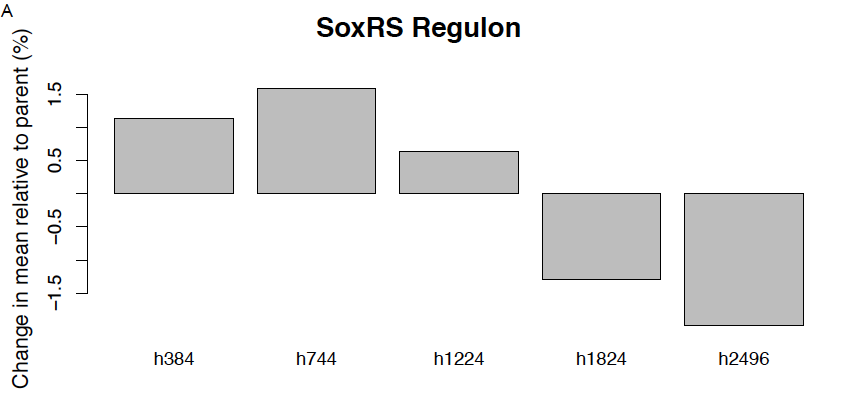


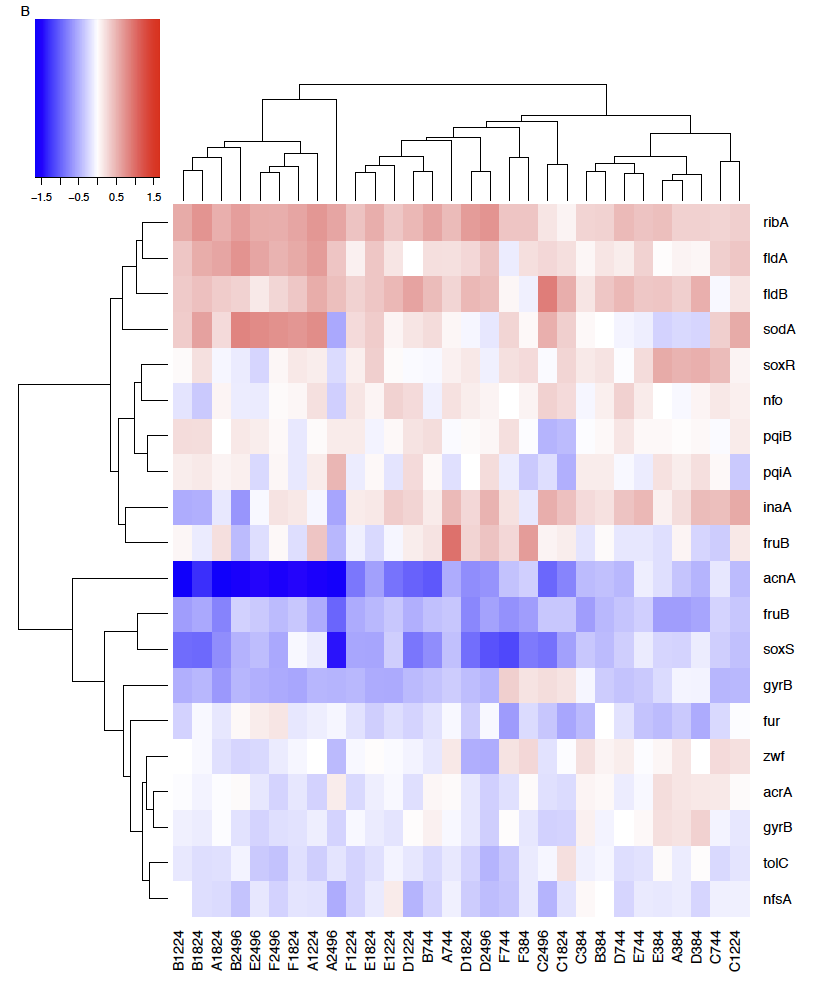


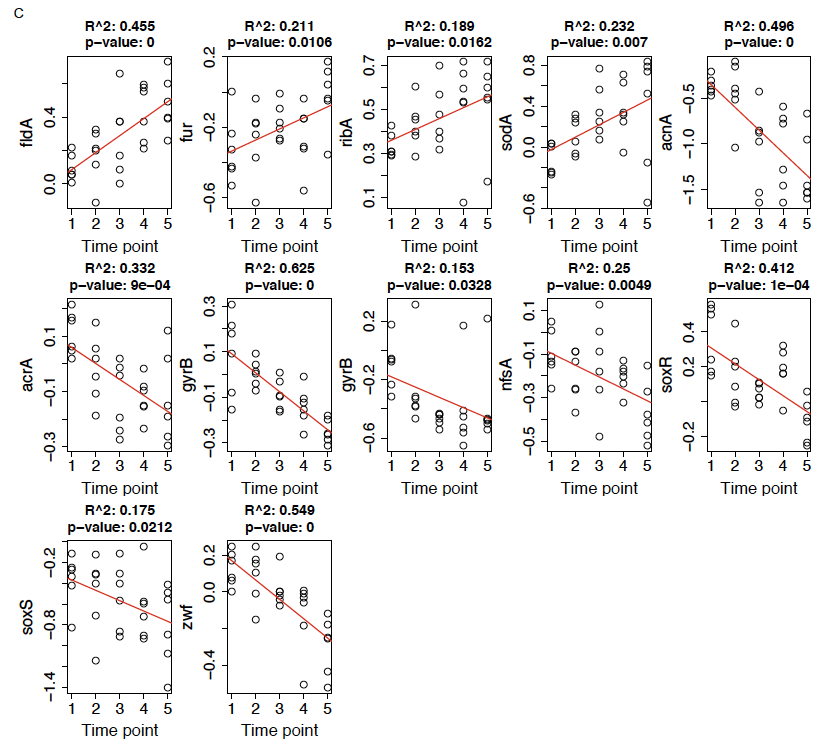


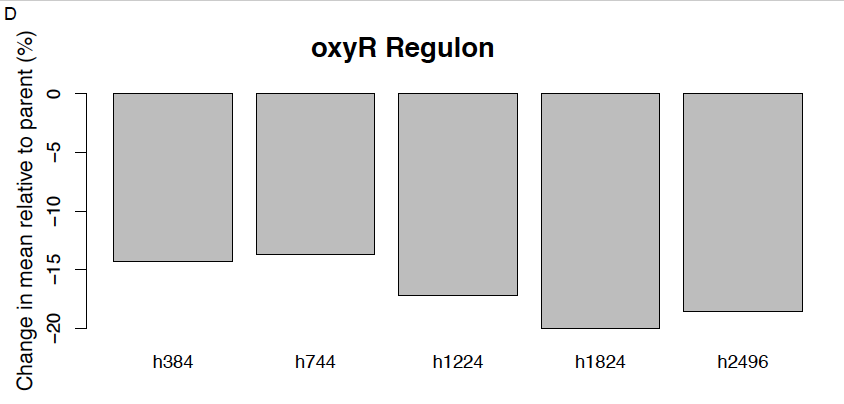


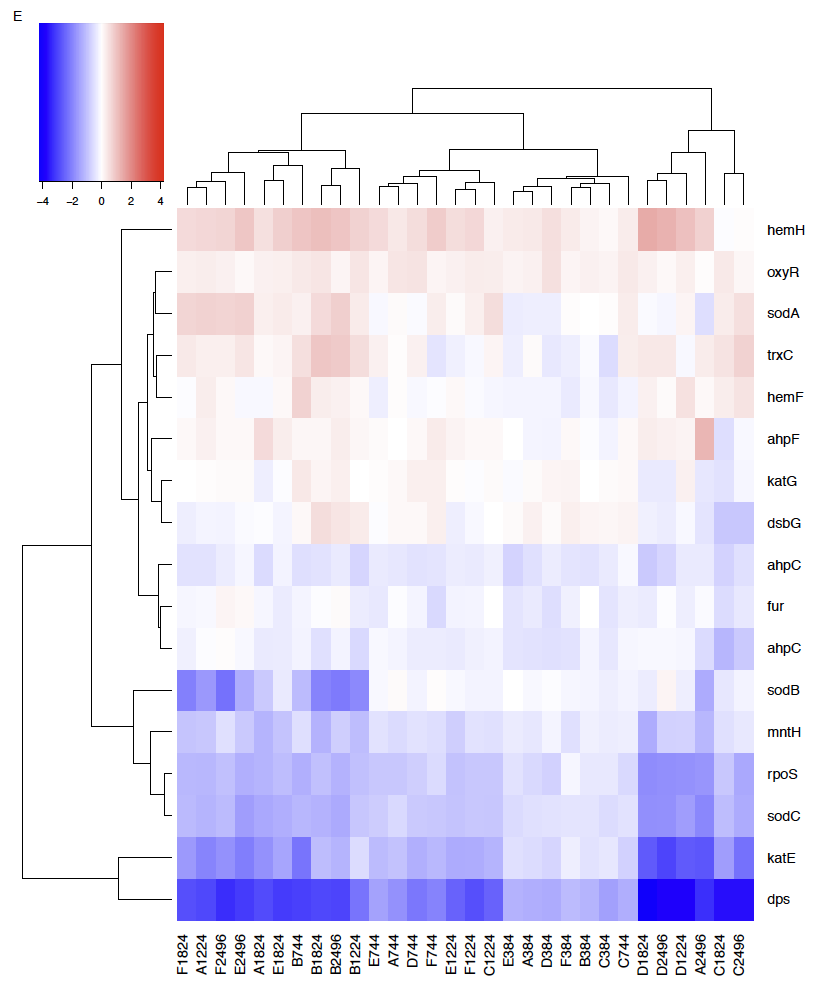


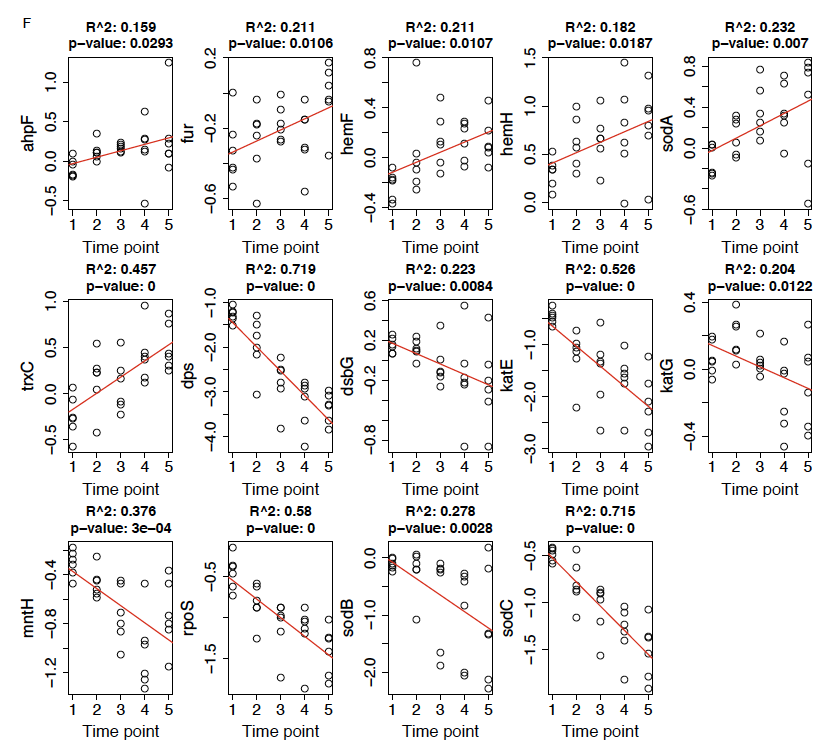


**Supplementary Fig. S13. Consistent downregulation of oxygen-sensitive *nrdD* in six EA strain during evolution. *y*-Axis represents the log2(fold change) relative to parent strain. *x*-axis represents five time points.**


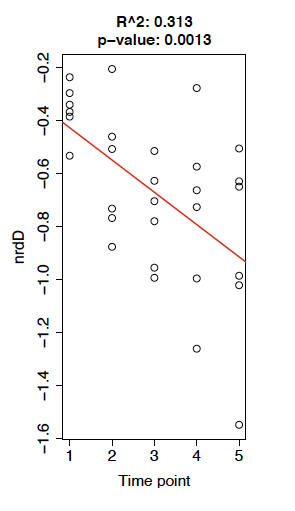


**Supplementary Fig. S14. Consistent downregulation of genes in anaerobic respiration in the EA samples.**


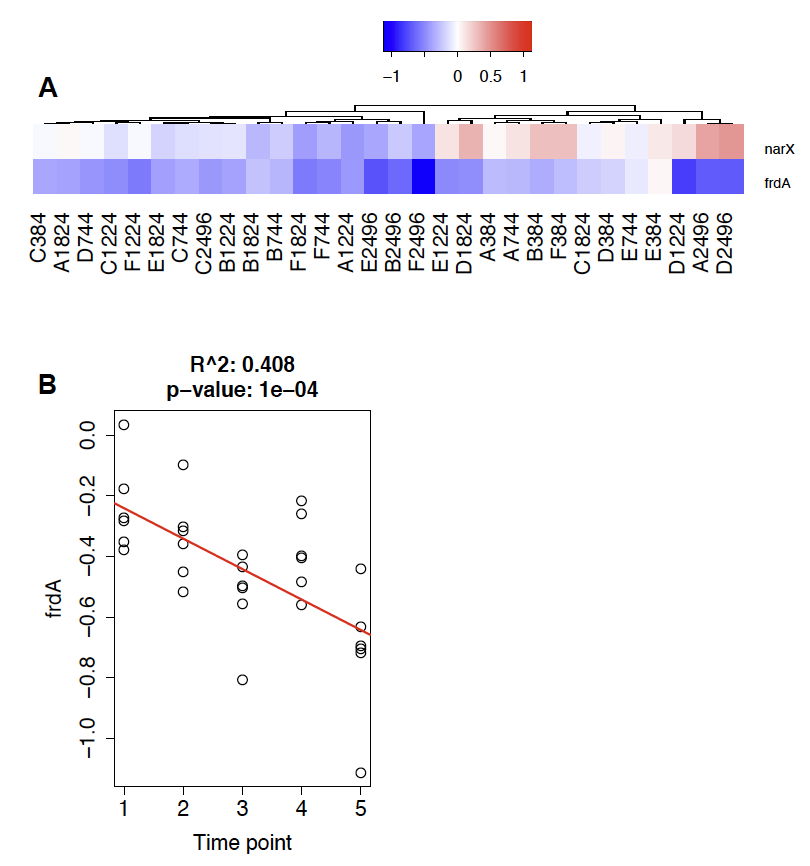


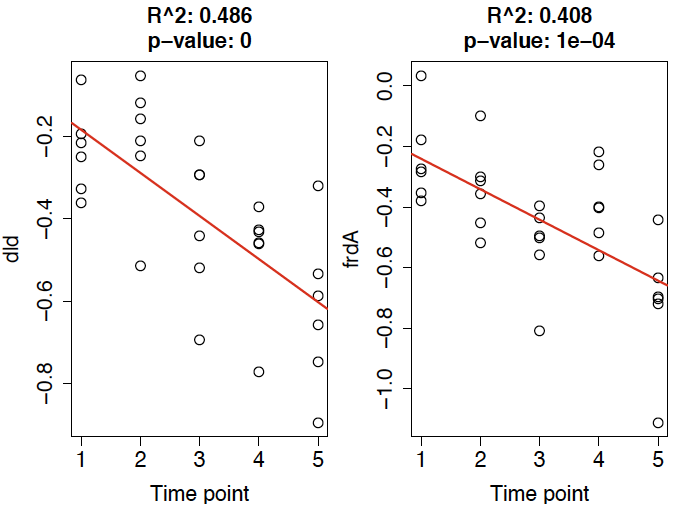


**Supplementary Fig. S15. Downregulation of genes in fermentation in EA samples during evolution.**


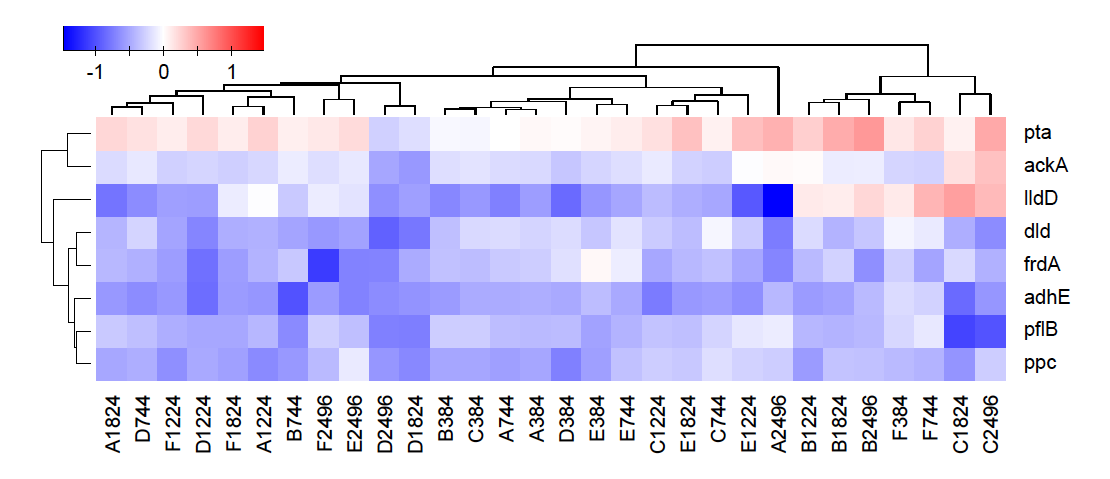


**Supplementary Fig. S16. The only two genes of fatty acid oxidation that are expressed and downregulated in the EA samples.**


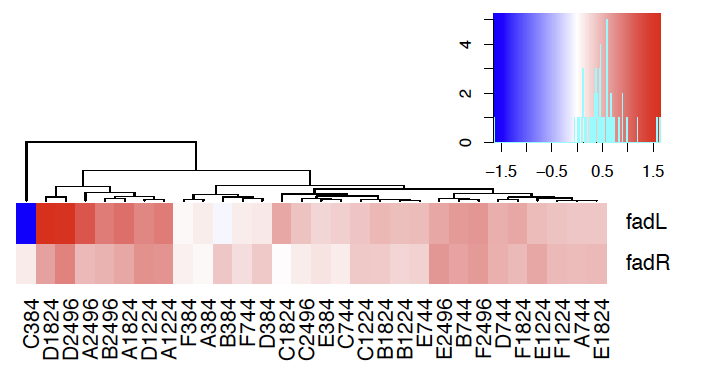


**Supplementary Fig. S17. Differential regulation of PTS genes along evolution in EA samples (A) and seven progressively downregulated PTS genes during evolution (B).**


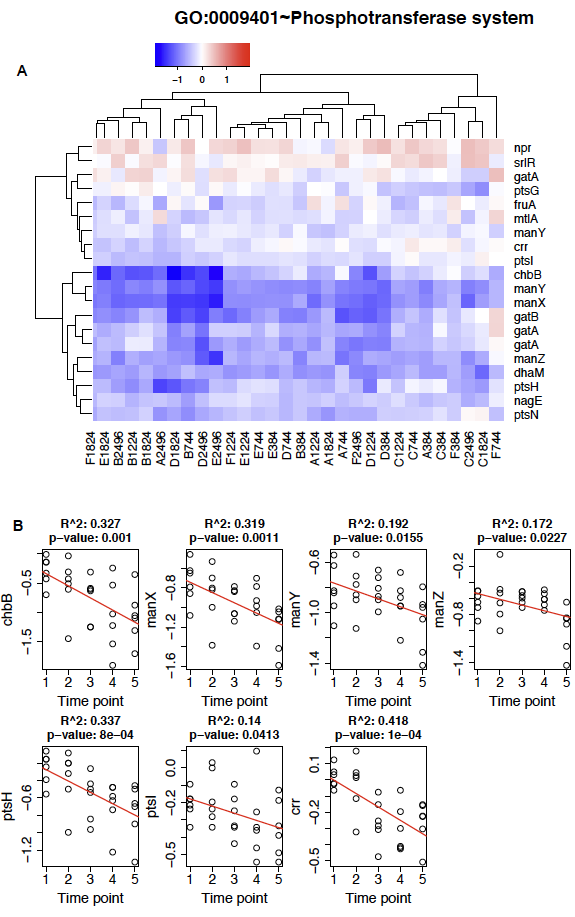


**Supplementary Fig. S18. Differential expression of genes in menaquinone biosynthesis in EA samples. A: differentially regulated genes; B: progressively downregulated genes.**


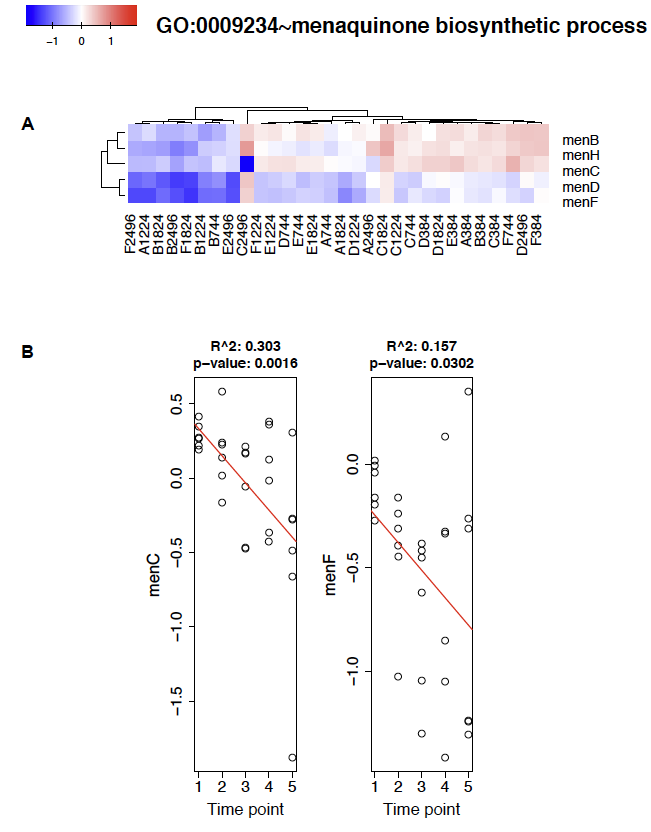


**Supplementary Fig. S19. Differentially expressed genes involved in peptidoglycan biosynthesis at three stages in the EA samples. A: initiation stage; B: crosslinking stage; and C: maturation stage. *x*-Axis indicates six strains (A-F) at five time points (384, 744, 1224, 1824, and 2496) and *y*-axis are genes involved in aerobic respiration.**


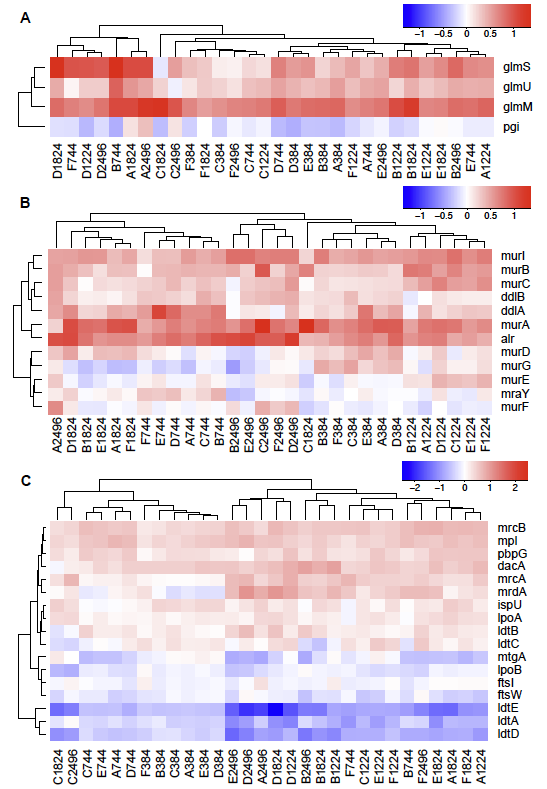


**Supplementary Fig. S20. The proportion of aberrantly-expressed TUs regulated by two activating EBP TFs in EA samples.**


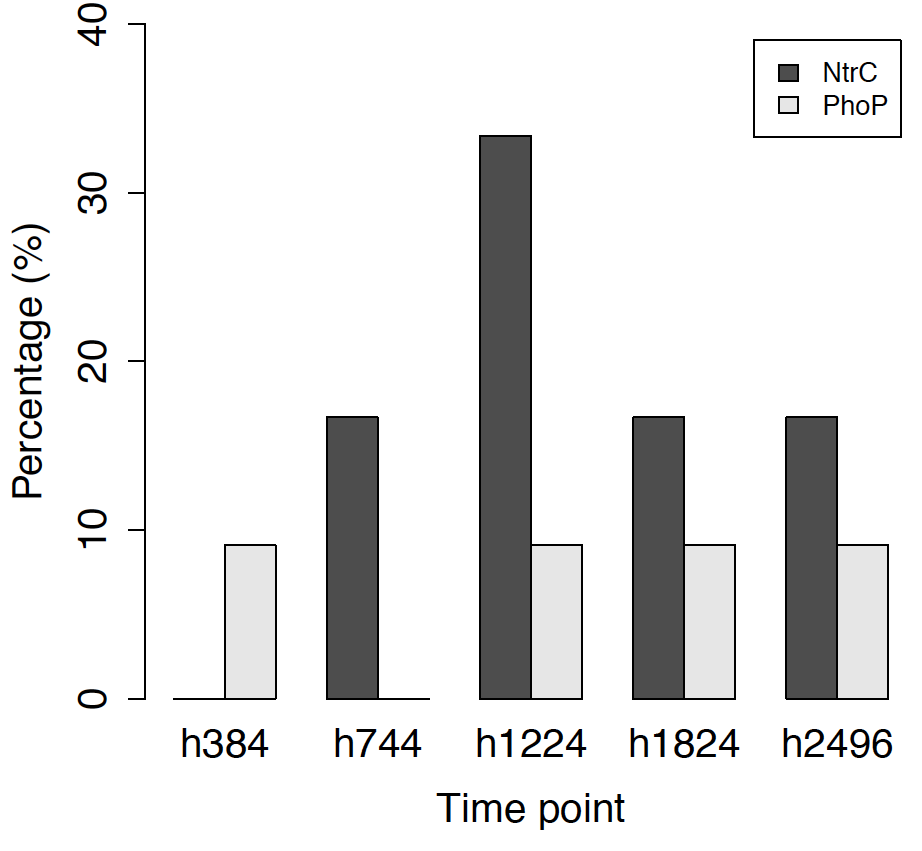


**Supplementary Fig. S21. Differential expression of genes in general stress response in EA samples over evolution. A: heat map; B: gene expression level over time.**


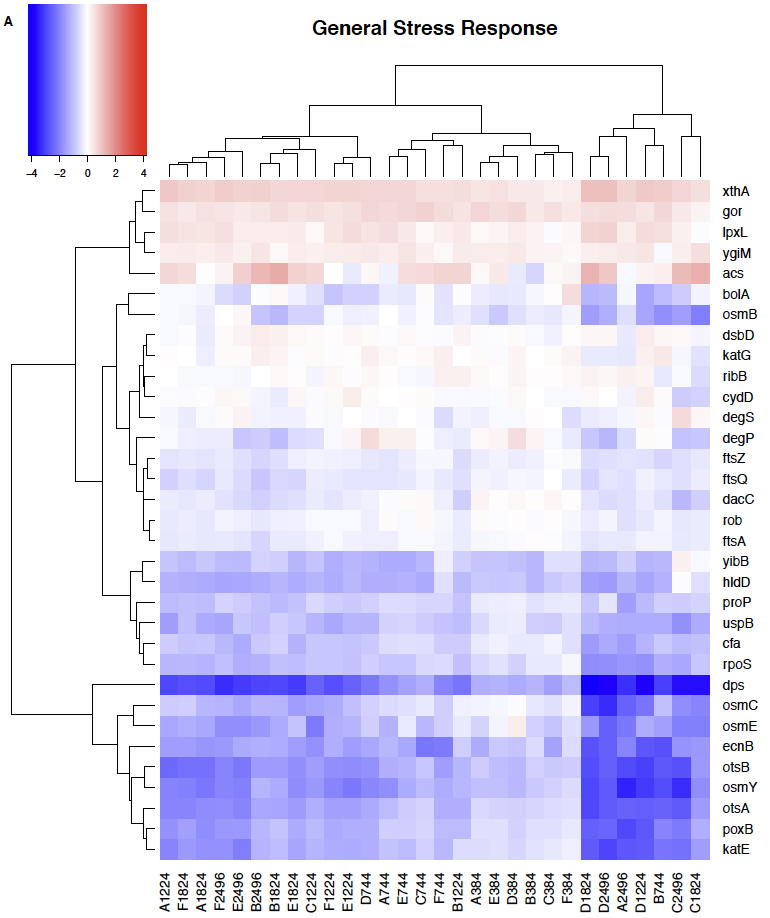


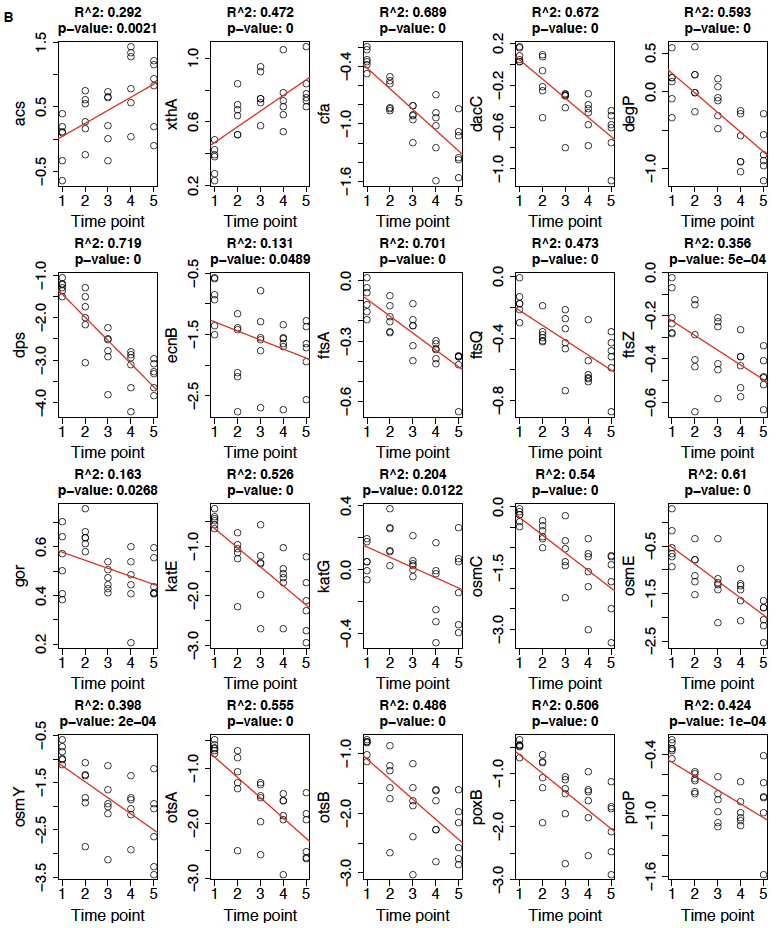


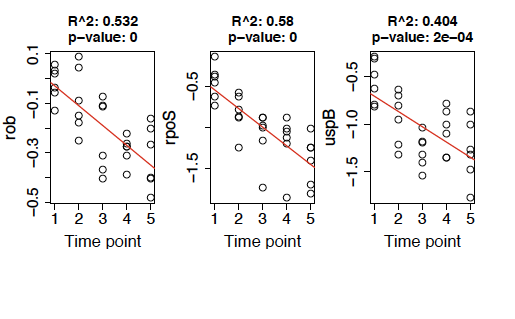


**Supplementary Fig. S22. The progressive upregulation of *hns* (A) and the differential expression of *hns* regulon in the six EA strains (B). (C) genes show step-wise upregulation during evolution; and (D) genes show step-wise decrease in evolution.**


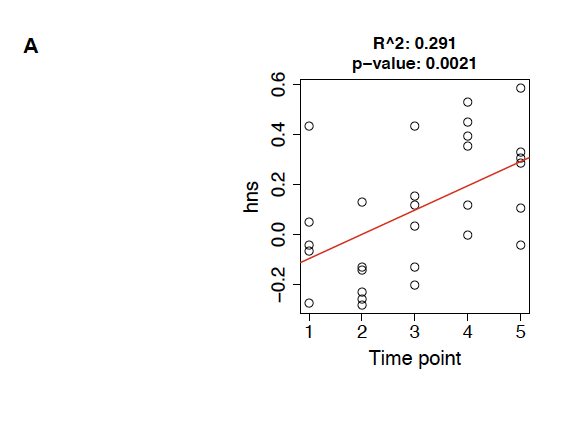


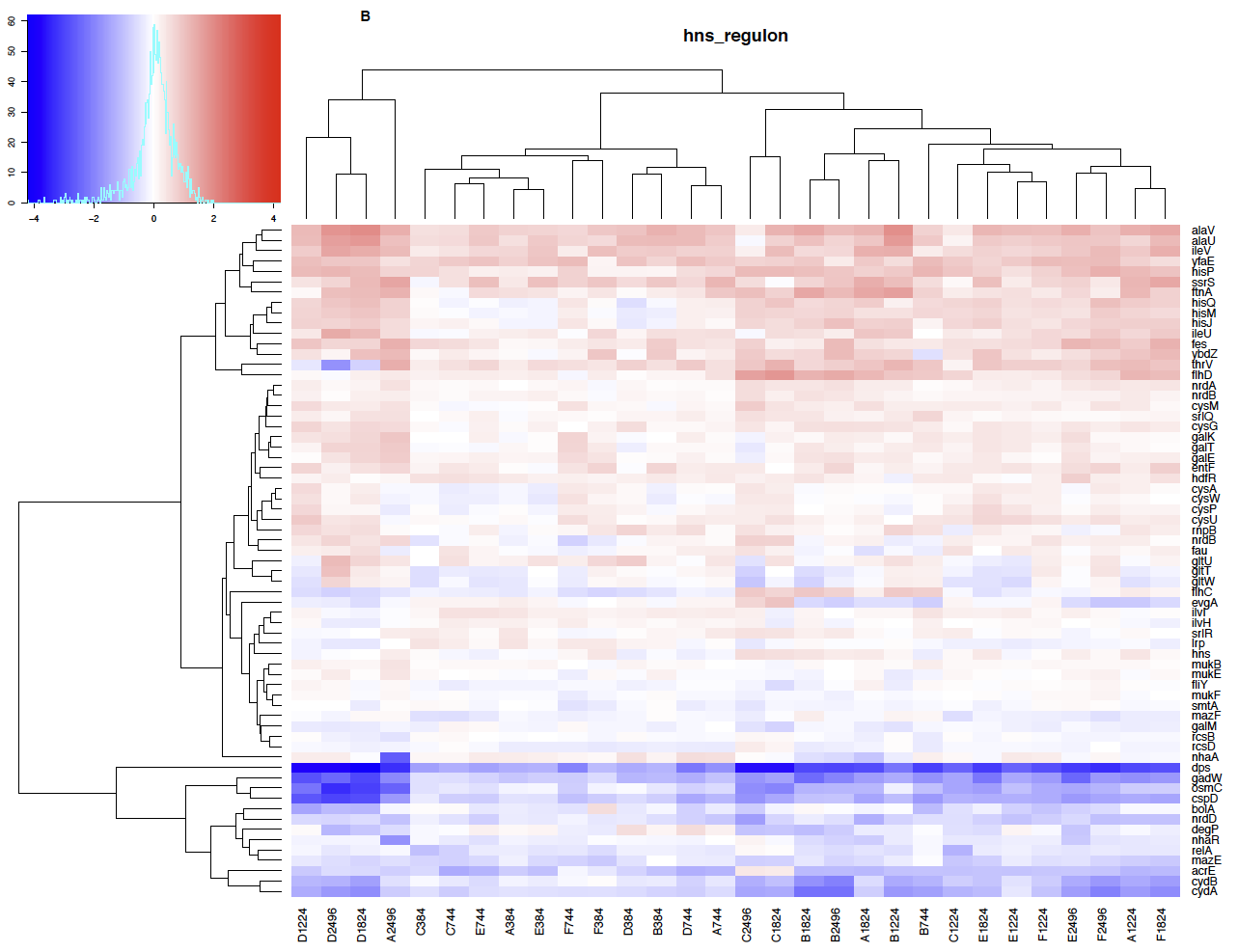


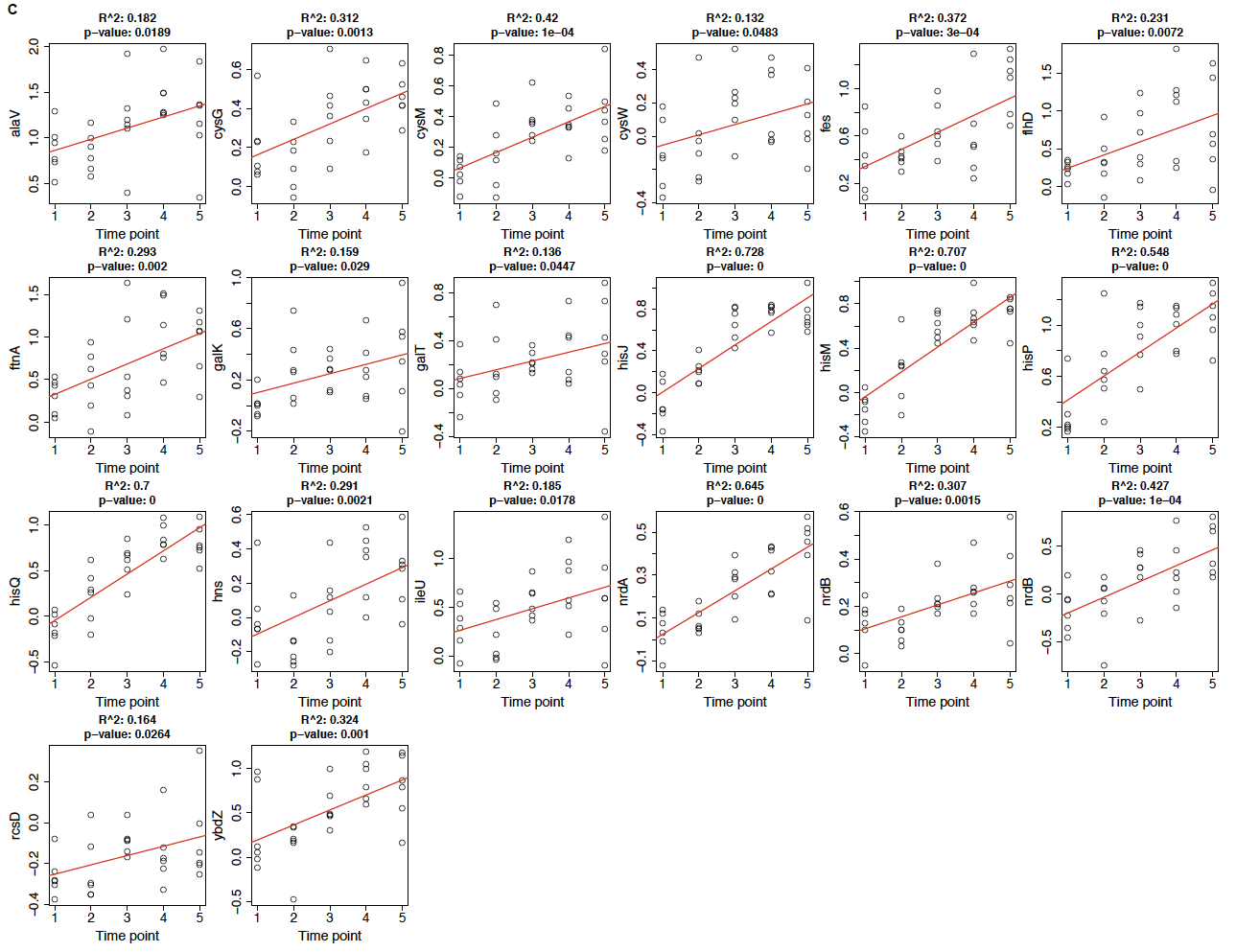


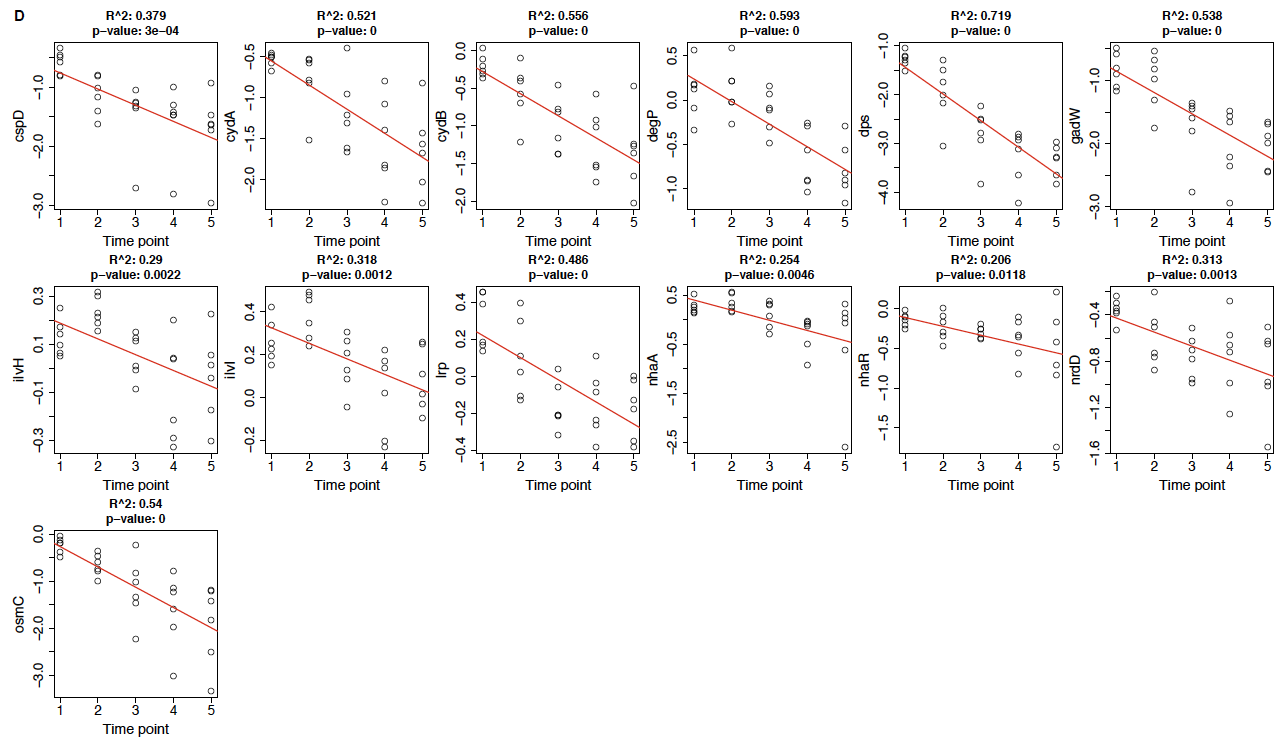


**Supplementary Fig. S23. Differential expression of genes in SOS response in six EA samples during evolution (A) and consistently upregulated SOS genes in six EA samples during evolution (B).**


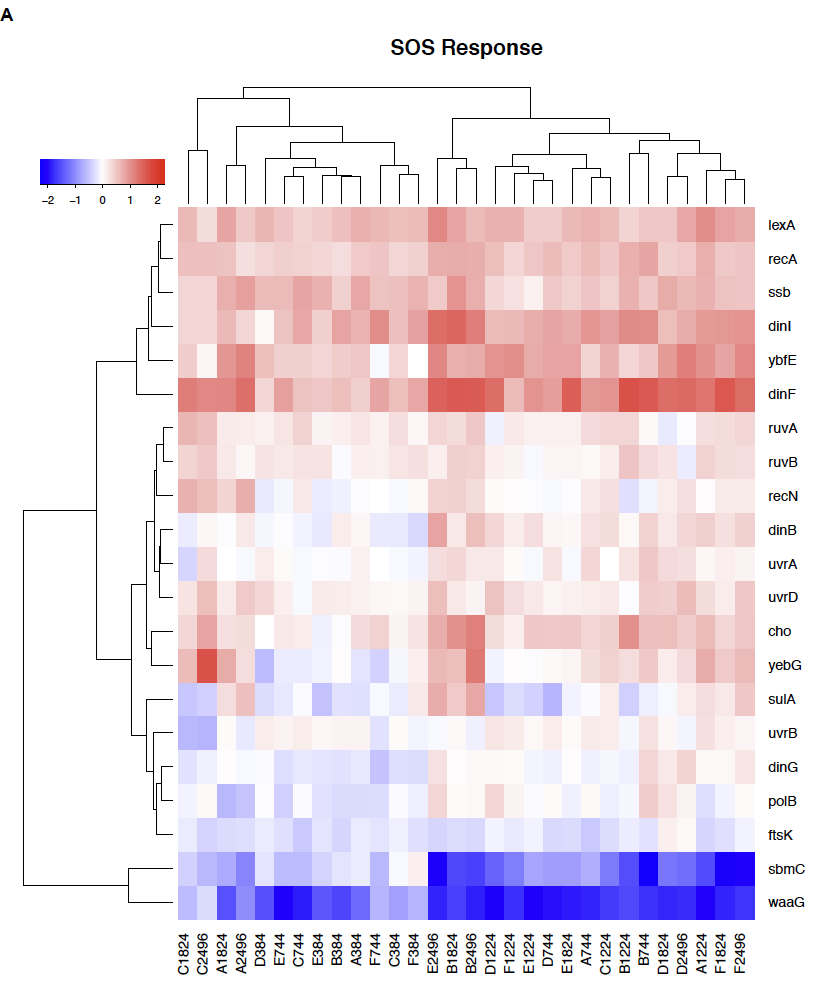


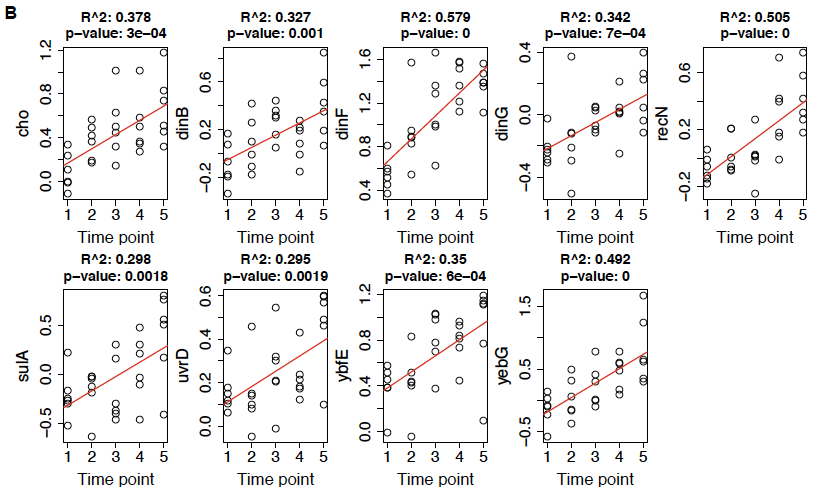


**Supplementary Fig. S24. Consistently upregulated genes in cold shock response in EA samples. (A) differential gene expression and (B) step-wise upregulation during evolution.**


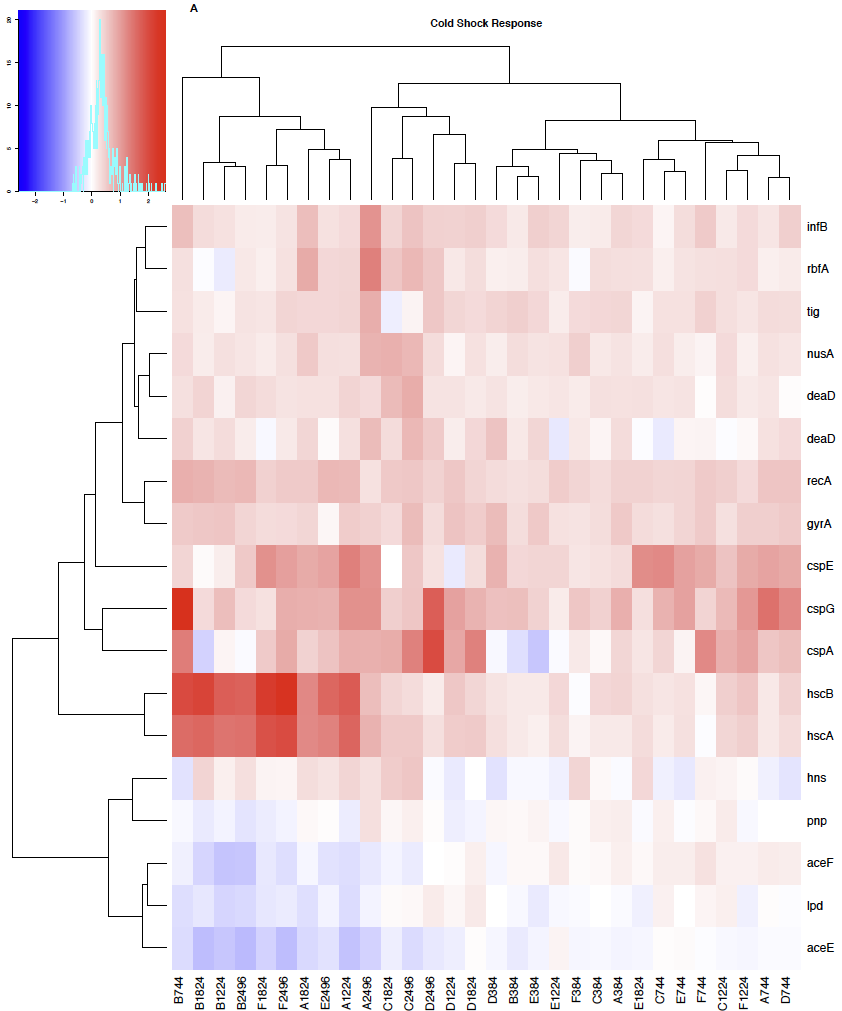


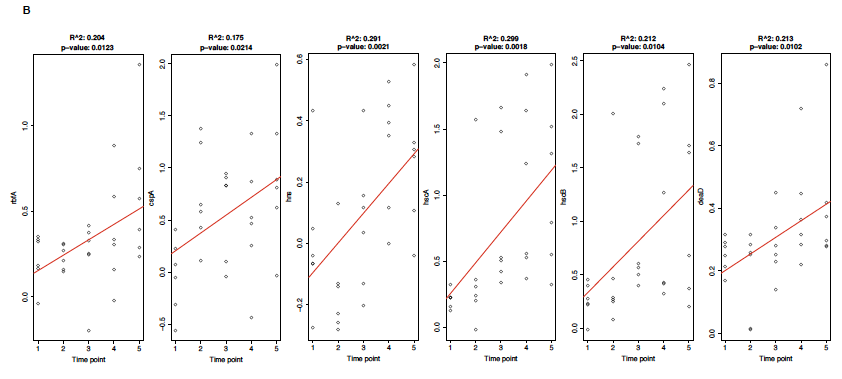


**Supplementary Fig. S25. The differential expression of genes in ribosomal biogenesis in EA samples.**


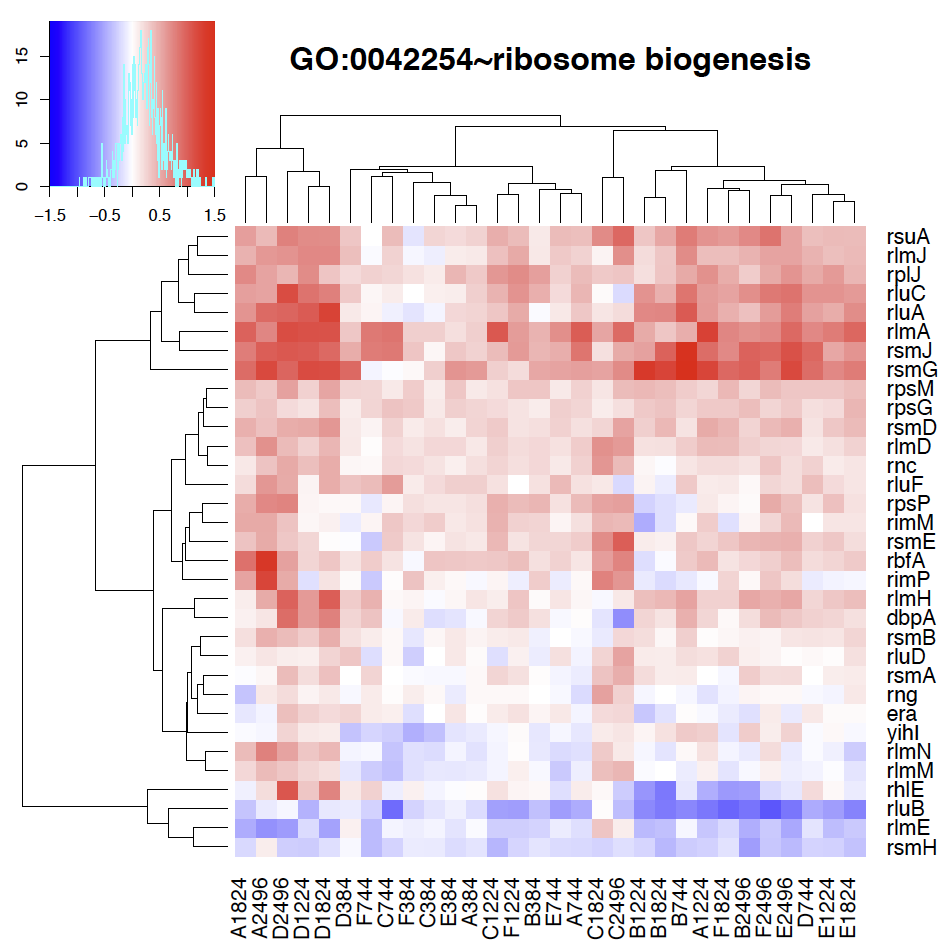


**Supplementary Fig. S26. Gene expression profiles of two present gene in fatty acid oxidation in the evolved strains. A: Differential expression; and B: consistent upregulation of two genes.**


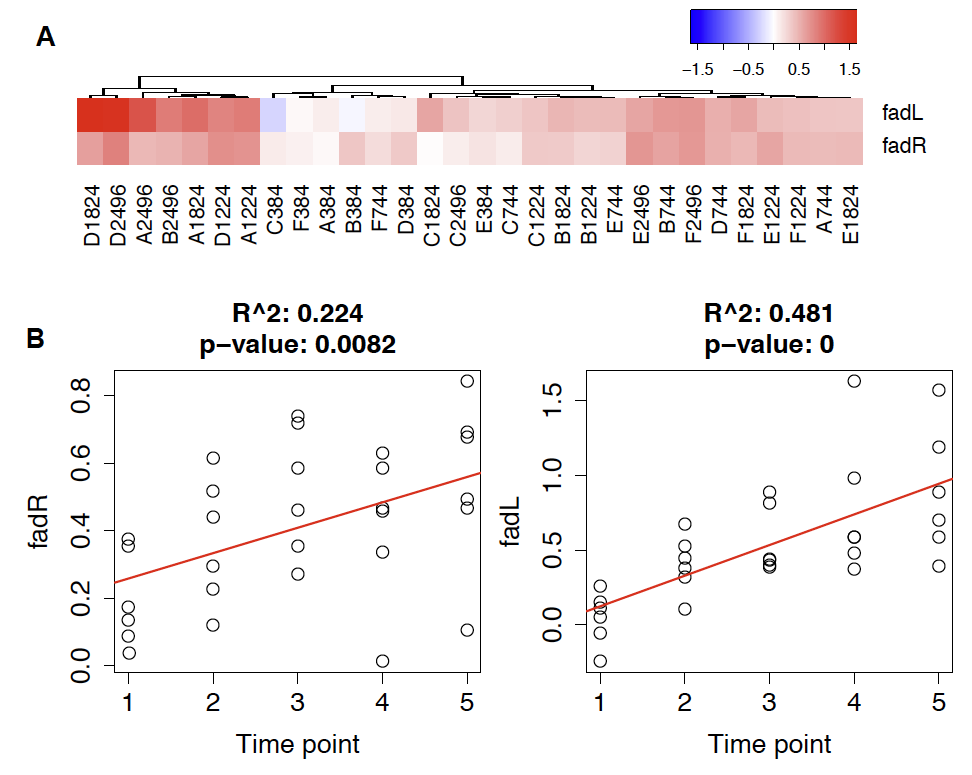


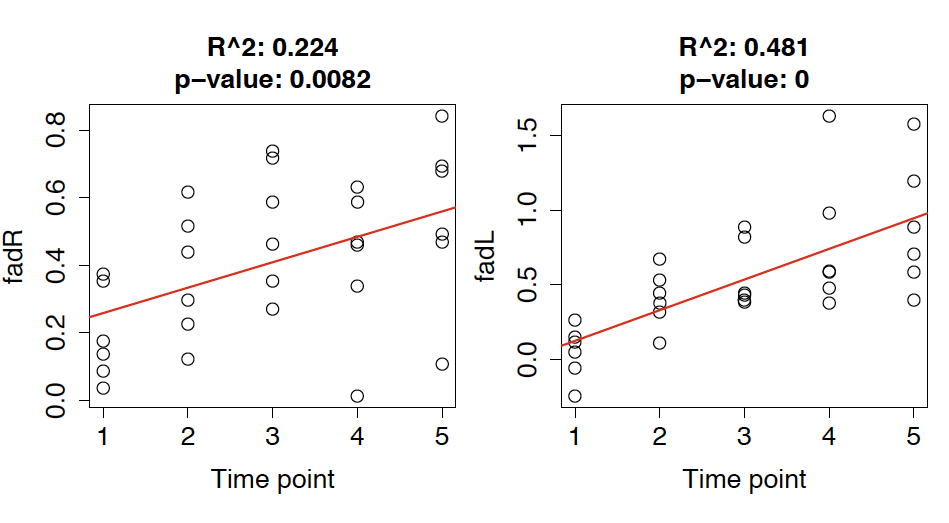


**Supplementary Fig. S27. Differential expression of genes involved in fatty acid biosynthesis in the EA samples.**


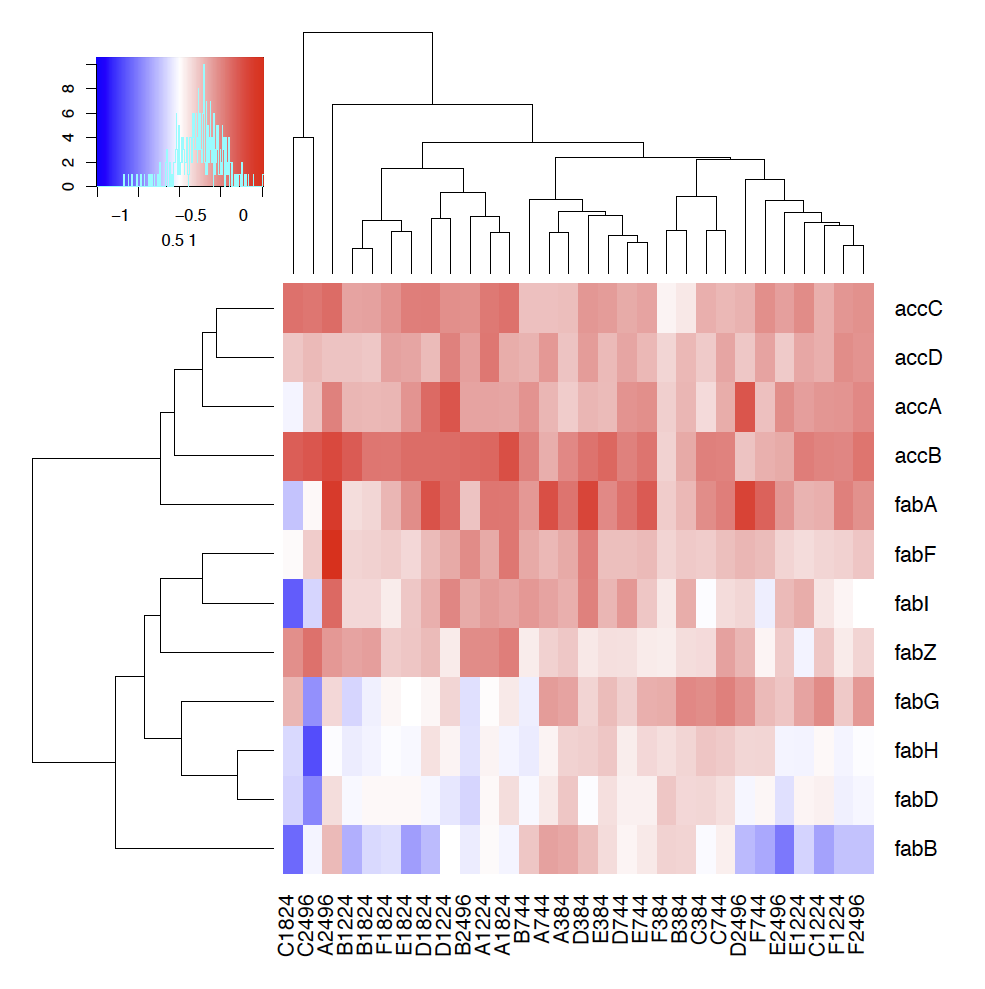


**Supplementary Fig. S28. Consistently upregulated gene involved in unsaturated fatty acid biosynthesis in the EA samples.**


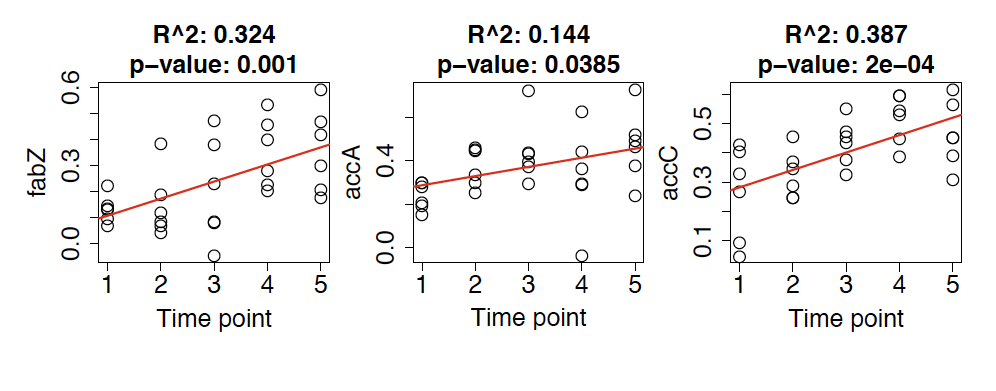


**Supplementary Fig. S29. Differential expression of genes involved in the incorporation of fatty acids into glycerolipids in the evolved strains. A: gene expression profiles; and B: consistently upregulated genes over time.**


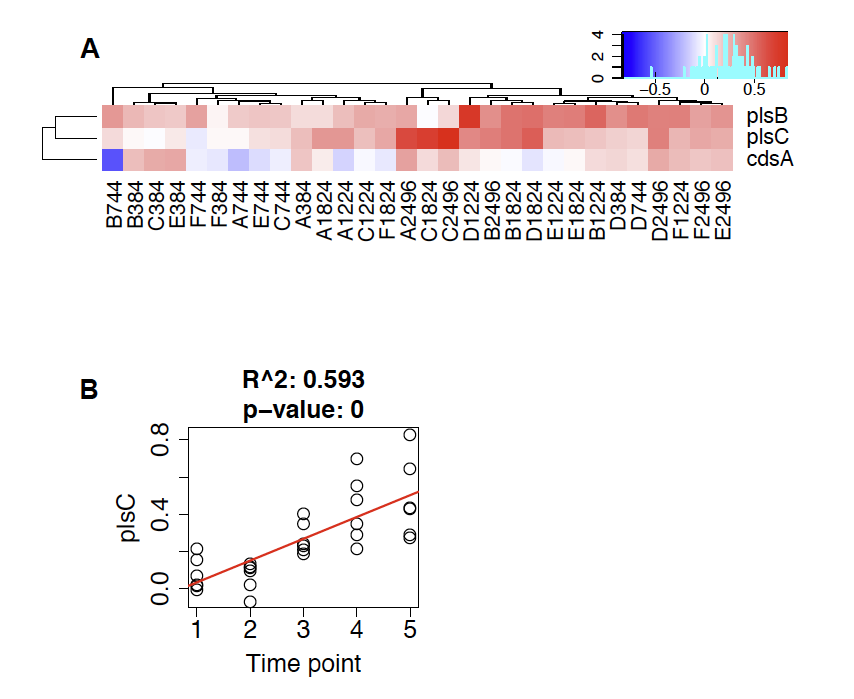

Supplement: Supplementary Information [file srep44150-s1.doc]
